# Immune Checkpoint Blockade Therapy May Be a Feasible Option for Primary Pulmonary Lymphoepithelioma-like Carcinoma

**DOI:** 10.3389/fonc.2021.626566

**Published:** 2021-04-26

**Authors:** Zuohong Wu, Xinghong Xian, Ke Wang, Deyun Cheng, Weimin Li, Bojiang Chen

**Affiliations:** ^1^ Department of Respiratory and Critical Care Medicine, West China Hospital of Sichuan University, Chengdu, China; ^2^ West China School of Medicine, Sichuan University, Chengdu, China

**Keywords:** lung cancer, primary pulmonary lymphoepithelioma-like carcinoma, treatment, immune checkpoint inhibitors, immunotherapy

## Abstract

Primary pulmonary lymphoepithelioma-like carcinoma (PPLELC) is a rare subtype of non-small cell lung cancer (NSCLC) for which there is currently no recognized treatment. Recently, favorable immune checkpoint blockade responses have been observed in PPLELC. This study aimed to review the effects of this regimen in patients with advanced PPLELC. PPLELC patients treated with immune checkpoint inhibitors at West China Hospital between January 2008 and December 2019 were retrospectively identified. Demographic parameters and antitumor treatment details were retrieved and reviewed. Among 128 patients diagnosed with PPLELC, 5 who received immune checkpoint inhibitors at advanced stages were included in the analysis. All of these patients were female nonsmokers with a median age of 55.6 (range 53-58) years at diagnosis. Their median PD-L1 expression was 40% (range, 30-80%). Although the patients underwent surgeries, chemotherapy and radiotherapy, all the treatments failed. Immune checkpoint inhibitors were administered palliatively, and three patients responded favorably, with the best overall response being partial remission (PR). Thus, immune checkpoint inhibitors may be a promising treatment for advanced PPLELC, and large clinical trials are warranted to obtain more evidence regarding this regimen.

## Introduction

Primary pulmonary lymphoepithelioma-like carcinoma (PPLELC) is a rare subtype of non-small cell lung cancer (NSCLC) that was first described by Begin et al. in 1987 (1). In the latest 2015 World Health Organization classification, PPLELC is categorized under “other and unclassified carcinomas” (2). Due to its rarity, the reported cases of PPLELC mainly occur in Southeast Asia and are believed to be associated with Epstein-Barr virus (EBV) infection (3).

Programmed cell death-1 (PD-1) is a member of the B7 family that is expressed by activated T cells along with its ligand, i.e., programmed death-ligand 1 (PD-L1), to mediate immunoregulation (4). PD-L1 is another immune checkpoint cell-surface protein that is expressed by tumor cells and host cells (5). The interaction between PD-1 in T cells and PD-L1 in tumor cells leads to inhibition of the proliferation of activated T cells (6). Thus, the inhibition of this interaction *in vivo* contributes to the enhancement of T-cell responses and can have antitumor activity (7).

Prior studies have shown higher than average expression of PD-L1 in PPLELC, which is also high compared with that in conventional NSCLCs (8, 9). Therefore, the high expression of PD-L1 in PPLELC suggests the potential benefit of using immunotherapy in this subtype of lung cancer. Currently, there has been no recognized treatment for PPLELC. Most patients diagnosed with PPLELC often present in early stages, and complete resection is performed (10). However, for advanced cases, multimodal therapy, including systematic chemotherapy and radiotherapy, is often needed (11). Recently, immune checkpoint inhibitors have emerged as treatment targets for NSCLCs, and favorable treatment responses against PPLELC have been reported (12–14).

In the present study, we enrolled patients with advanced PPLELC who underwent immune checkpoint blockade therapy with the aim of reviewing our preliminary experience with the use of this regimen in patients with advanced PPLELC.

## Materials and Methods

This retrospective study included patients with histologically confirmed PPLELC at West China Hospital between January 2008 and December 2019. The patients were identified through hospital pathological and medical electronic databases, and records regarding demographic parameters, clinical manifestations, laboratory test results, chest computed tomography (CT) features, diagnostic methods, antitumor treatment and treatment reactions were simultaneously retrieved. All eligible patients received immunotherapy as the treatment for PPLELC and were followed up until June 30, 2020.

The pathological diagnosis of PPLELC was based on a combination of hematoxylin-eosin (HE) and immunohistochemical (IHC) staining and Epstein-Barr encoding region (EBER) positivity of lung tissue resections, and all patients underwent CT, magnetic resonance imaging (MRI), or positron emission tomography (PET)/CT to rule out nasopharyngeal cancer or lymphoepithelioma-like carcinoma (LELCs) of other origins. The tumor staging classification was based on the tumor-node-metastasis staging system (15). The expression level of PD-L1 was detected by immunohistochemistry using anti‐PD‐L1 antibody (clone 28-8, ab205921, Abcam). The results are expressed as a tumor proportion score (TPS), indicating the percentage of viable tumor cells showing partial or complete membrane staining at some intensity in the tissue specimens, i.e., TPS of 0–1% was regarded as negative, 1%–49% as low and ≥ 50% as high expression (16). We adopted the Response Evaluation Criteria in Solid Tumors (RECIST) version 1.1 to assess changes in the tumor burden (17).

## Results

### Patient Characteristics

**Figure 1:**
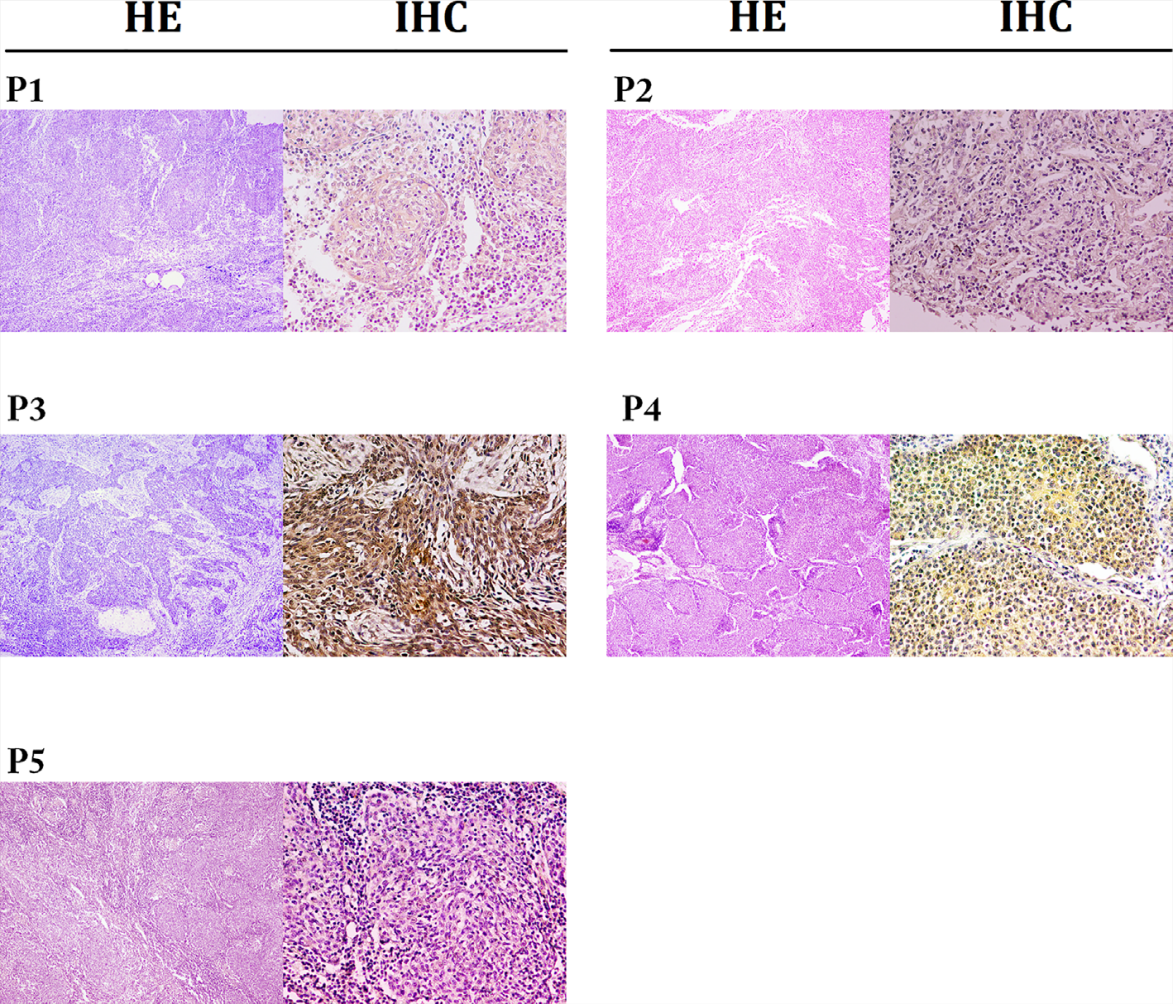
Representative images for HE (×40) and IHC staining for PD-L1 expressions (×200). The expressions of PD-L1 in patients 1 to 5 were 40%, 30%, 90%, 80% and 5%, respectively.

In total, we screened 128 patients diagnosed with PPLELC, including 5 who received immunotherapy. The demographic characteristics of these 5 patients are displayed in **Table 1**. All of these 5 patients were female nonsmokers with a median age of 55.6 (range 53-58) years at diagnosis. Furthermore, almost all of them had a tumor size greater than 3 (median 5.1, range 4.7-6.4) cm. In two patients, the tumors were located in the right middle lobe; in the other 3, the tumors were in the right lower lobe, left upper lobe and left lower lobe. The stage distribution at initial diagnosis was IA in one patient, IIIA in one patient and IV in three patients. Moreover, three patients had a PD-L1 TPS of less than 50% (case 1, 40%; case two, 30%; case 5, 5%), and two patients had a PD-L1 TPS of more than 50% (case 3, 90%; case 4, 80%). Two patients showed evidence of EBV infection, and the overall TPS of PD-L1 was 40% (range, 30-80%), including two patients with high expression (≥50%) and three with low expression (5-49%). Notably, one patient (case 3) was misdiagnosed with pulmonary squamous cell carcinoma before her biopsy samples were sent to the Pathology Department of our hospital for consultation. Representative images of the HE and IHC staining of PD-L1 expression are shown in **Figure 1**.

**Table 1:** Demographic characteristics of the patients with PPLELC.

Patient	Sex	Age (y)	Smoking status	Method of diagnosis	Site of tumor	Tumor size (cm)	TNM staging	Overall staging	Serum EBV examination	PD-L1 expression	*EGFR*	*ALK*	*ROS*-1
**1**	Female	58	N	Operation	RLL	5.1	T3N2M0	IIIA	unknown	40%	Negative	Negative	Negative
**2**	Female	53	N	Operation	LUL	2.3	T1bN0M0	IA2	unknown	30%	Negative	Negative	Negative
**3**	Female	48	N	EBUS bronchoscopy	RML	4.7	T2bN2M1	IV	unknown	90%	Negative	Negative	Negative
**4**	Female	56	N	CT-guided percutaneous needle lung biopsy	LLL	6.4	T4N2M1b	IV	EBV-EA-IgG positive	80%	Negative	Negative	Negative
**5**	Female	63	N	Bronchoscope biopsy	RML	7.0	T4N3M1	IV	EB-DNA 9.10E+03 copies/mL	5%	Negative	Negative	Negative

EBUS, endobronchial ultrasound; RLL, right lower lobe; LUL, left upper lobe; RML, right middle lobe; LLL, left lower lobe; EBV, Epstein-Barr virus; PD-L1, programmed cell death-1.

### Treatment Before Immune Checkpoint Blockade Therapy

The treatment details before application of immune checkpoint inhibitors are shown in **Table 2**. Only two patients underwent radical tumor resection, and adjuvant chemotherapy and gemcitabine plus cisplatin (GP regimen) were administered to Patient 1. Progression-free survival (PFS) in the first two patients was 19.4 months and 8.3 months, respectively. Palliative chemotherapies, including paclitaxel plus carboplatin (TC regimen), pemetrexed (Alimta) plus carboplatin (AC regimen), docetaxel plus cisplatin (DP regimen) and paclitaxel plus fluorouracil (TF regimen), were administered to all patients at advanced stages, and the median number of chemotherapy cycles given to them was 1. The first four patients received the TC regimen only and achieved stable disease (SD). The fifth regimen was administered sequentially to the AC, DP, and TF regimen patients and resulted in partial remission (PR). Additionally, three patients received thoracic radiotherapy. However, all patients ultimately had progressive disease (PD), and the median time to first tumor progression was 7.4 (range, 5.2-9.6) months.

**Table 2:** Treatment details before immune checkpoint blockade therapy.

Patient	Surgery	Adjuvant chemotherapy	PFS since surgery (m)	No. of chemotherapy regimens	Chemotherapy regimens	PFS since following palliative chemotherapy (m)
**1**	Yes	Yes (GP)	19.4+	1	TC	15.7+
**2**	Yes	No	8.3	1	TC	10.6
**3**	No	No	NA	1	TC	5.6
**4**	No	No	NA	1	TC	4.2
**5**	No	No	NA	3	AC, DP, TF	9.3*, 9.7**

GP, gemcitabine plus cisplatin; PFS, progression-free survival, NA, not applicable; TC, paclitaxel plus carboplatin; AC, pemetrexed (Alimta) plus carboplatin; DP, docetaxel plus cisplatin; TF, paclitaxel plus fluorouracil; SD, stable disease; PR, partial remission.

^*^PFS1 with AC regimen; ^**^PFS2 with DP regimen.

### Immune Checkpoint Blockade Therapy

**Figure 2:**
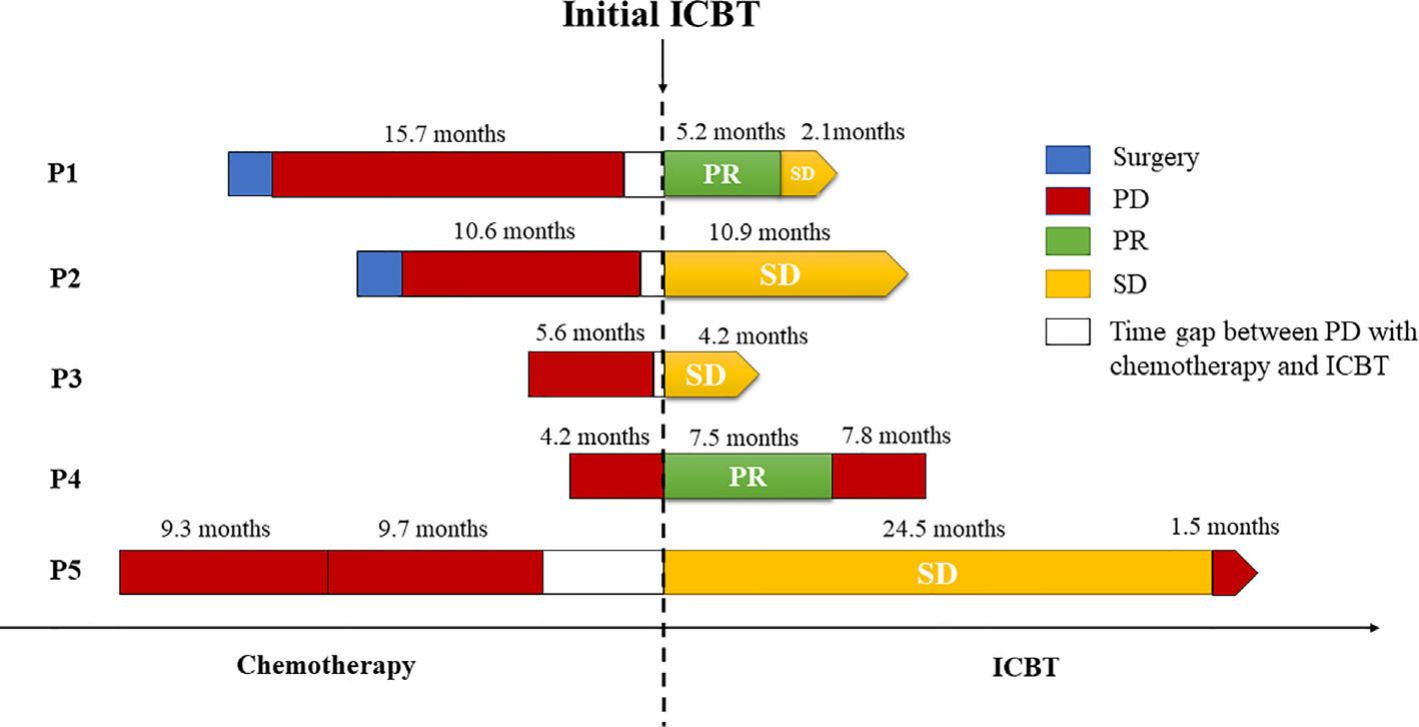
Summary of treatment reactions to chemotherapy and immune checkpoint blockade therapy. All of the patients underwent PD before immunotherapy, and three of them responded favorably to ICBT. The bars on the left and right side of the dotted line denote the treatment details before and after the administration of immune checkpoint inhibitors, respectively.

**Table 3** presents details on the immunotherapy regimen. In total, three types of immune inhibitors were used in our patients, including sintilimab, pembrolizumab and nivolumab. These five patients underwent a median of 8.8 (range, 5.5-14.7) months of palliative chemotherapy and/or radiation before immunotherapy was adopted. The median number of immunotherapy cycles administered to our patients was 8 (range, 6-19), and the best treatment response achieved was PR in two patients and SD in three patients. At the end of the final month, two of our patients had developed PD. Our second patient developed PD in the initial four cycles of pembrolizumab alone, but SD was subsequently achieved once she received pembrolizumab combined with nab-paclitaxel. We were unable to contact Patient 3 to obtain more information regarding her treatment details and subsequent status. Briefly, Patient 4 showed PD in the second follow-up after partial remission with a PFS following ICBT of 7.5 months. Unfortunately, she died 7.8 months after progression. Compared with the PFS of 4.2 months following chemotherapy, survival for 15.3 months following ICBT may be considered an improvement. Patient 5 also experienced PD in the final month (in May 2020), with SD for 24.5 months. A stable period of 24.5 months following ICBT was a significant improvement over that resulting from the previous chemotherapy, especially because she suspended ICBT for more than 9 months due to financial reasons. Fortunately, both Patient 1 and Patient 2 continued to benefit from ICBT without progression. A summary of the overall treatment reaction is presented in **Figure 2**.

**Table 3:** Treatment details of immune checkpoint blockade therapy.

Patient	Time gap between ICBT and chemotherapy(m)	PD-L1 expression	ICBT	Cycles of ICBT received	Best overall response	PD to ICBT	PFS with ICBT	Duration of following the start of ICBT (m)	Living status	Survival from the start of ICBT (m)	Survival from the start of chemotherapy(m)
**1**	17.5+	40%	Sintilimab+Anlotinib	8 (ongoing)	PR	No	NA	8.3	Alive	8.3	25.8
**2**	11.6	30%	Pembrolizumab+nab-paclitaxel	6 (ongoing)	SD	No	NA	10.9	Alive	10.9	22.5
**3**	6	90%	Pembrolizumab	1	SD	UN	UN	4.2+	UN	4.2+	10.2+
**4**	4.1	80%	Nivolumab	19	PR	Yes	7.5	15.3	Dead	15.3	19.4
**5**	24.2	5%	Nivolumab+Anlotinib	21 (ongoing)	SD	Yes	24.5^*^	26.0	Alive	26.0	50.2

ICBT, immune checkpoint blockade therapy; PD-L1, programmed cell death-1; PD, progressive disease; PR, partial remission; SD, stable disease; UN, unknown.

^*^This patient had PD during the final month, with a PFS of 24.5 months, during which she suspended ICBT for more than 9 months due to hypothyroidism and financial reasons.

Images of the changes observed throughout treatment are displayed in **Supplementary Figures 1–5**, and additional information for the excluded 123 patients is presented in **Supplementary Table 1**.

## Discussion

We reviewed the efficacy of immune checkpoint inhibitors in patients with PPLELC, and based on our preliminary experience, most patients responded favorably to the PD1/PD-L1 blockade therapy. To the best of our knowledge, this study is the first to thoroughly summarize the treatment response of PPLELC patients to different checkpoint inhibitors.

Our patients had a median age of 55.6 years (range 53-58) and were predominantly female. The patients were all nonsmokers, which is quite a distinctive demographic characteristic of PPLELC, as it generally affects younger Asian nonsmoking females (18). The features of PPLELC that distinguish it from other subtypes of NSCLC indicate that PPLELC is a unique subtype of lung cancer. In addition, epidemiological differences in PPLELC exist across different regions worldwide (18, 19). As reported by other studies (13, 20, 21), PPLELC in our study mainly originated in the right lower lobe and rarely in the right upper lobe, with a median diameter of 5.1 cm. All of the patients had EBER positivity, and two showed evidence of EBV infection; although it is widely believed that a connection exists between EBV infection and the development of PPLELC (3), conflicting findings have been reported in Western populations (22). *In vitro* studies of nasopharyngeal carcinoma have shown that EBV has the ability to upregulate PD-L1 expression through IFN-γ and latent membrane protein 1 (18). The results of our study revealed a median PD-L1 TPS of 40%, and 60% of the patients had low PD-L1 expression, which is similar to the findings reported in the study by Zhanhong Xie et al. (23).

Patient 1 and Patient 2 first underwent radical tumor resection during early stages of the disease. In resectable cases, complete removal is the preferred approach to PPLELC. A study by Liang et al. suggested that a high survival rate can be achieved through radical resection (24). In their study, among 40 patients who underwent complete tumor resection, recurrence occurred in 6 patients, ranging from 10.6 to 41.1 months after the surgery. At the end of the final month of follow-up, these two patients were alive, with tumor progression times following surgery of 19.4 and 8.3 months. Notably, adjunct chemotherapy was administered to Patient 1, so whether longer progression-free survival could be achieved by adjuvant chemotherapy and/or radiation is unknown. Additionally, in Liang’s study, the authors concluded that in PPLELC patients with stage IIIA disease who underwent radical resection, a better prognosis could be achieved when adjuvant chemotherapy was administered (24). The results of a meta-analysis showed that NSCLC patients in stage IIIA benefited the most from adjuvant chemotherapy (25, 26). Therefore, postoperative adjuvant chemotherapy should be used for cases at locally advanced tumor stages. In addition, palliative chemotherapy and/or radiation are often used for patients in advanced or metastatic stages. In our study, the most commonly used chemotherapy was the TC regimen, and the corresponding patients achieved the best overall SD, regardless of the inclusion of radiotherapy in the treatment regimen. Currently, the optimal chemotherapy for PPLELC remains unclear. A study conducted in Macau compared the efficacy of taxane-based and non-taxane-based combinations, and the results did not show a significant difference in terms of response or survival (13). A retrospective study on Chinese Taiwan patients revealed that platinum-based doublet chemotherapy could be considered the first-line treatment for advanced PPLELC (27). Another study by Zuan Lin et al. assessed three first-line chemotherapy regimens, i.e., GP, taxanes plus platinum (TP) and pemetrexed plus platinum (AP); of these regimens, GP achieved the highest response rate and longest PFS (28). Furthermore, due to the uncertainty of adding thoracic radiotherapy to first-line chemotherapy, Zuan Lin et al. evaluated the value of radiotherapy and concluded that palliative thoracic radiotherapy was beneficial for prolonging the survival of PPLELC patients with advanced-stage disease (28). Consistent with our study, PFS was longer in Patients 1, 2, and 5, all of whom received thoracic radiotherapy.

Since our patients were all positive for PD-L1 expression, they received immunotherapy after systematic chemotherapy and radiotherapy either with or without concurrent treatments. The immune inhibitor administered to our first patient was sintilimab, and she achieved a response of PR. Initially approved for the treatment of classical Hodgkin’s lymphoma, sintilimab is a fully humanized IgG4 monoclonal antibody that binds PD-1 to block the interaction between PD-1 and its ligands (29). To date, our patient is the first worldwide to receive sintilimab as a palliative treatment for PPLELC, and we confirmed its efficacy. The latest clinical trial conducted in mainland China evaluated the safety and outcome of sintilimab as a neoadjuvant treatment in patients with resectable NSCLC (stage IA-IIIB). The results showed that sintilimab was well tolerated, and a 40.5% major pathological response was obtained (30). Our results suggest that the use of the PD-1 inhibitor sintilimab in advanced PPLELC patients may be feasible. Pembrolizumab and nivolumab are other PD-1 antibodies approved for the treatment of unresectable NSCLC. Although patient 2 developed PD on pembrolizumab alone in the initial four cycles, she still had SD when it was subsequently combined with nab-paclitaxel. Patient 3 was treated with 1 cycle of pembrolizumab and was able to achieve SD. A study by Na Zhou et al. described a female patient from Macau treated with pembrolizumab after a three-line platinum-combined chemotherapy regimen, and SD was achieved (13), representing the first report of a favorable response to pembrolizumab in patients with advanced LELC. In patients with advanced NSCLC and a PD-L1 TPS of 50% or more, the first-line treatment has been pembrolizumab monotherapy instead of platinum doublet chemotherapy (31).

Moreover, a multicenter retrospective study recently conducted by Aguilar EJ showed that the treatment effect of the first-line treatment pembrolizumab was significantly better in NSCLC patients with PD-L1 expression ≥ 50% than in those with PD-L1 expression ≥ 90% (32). Their findings suggest that higher PD-L1 expression in NSCLC patients may lead to better clinical outcomes for patients receiving pembrolizumab. This finding may account for the effect difference of pembrolizumab in our patients. Unfortunately, we were unable to maintain contact with Patient 3, who only received one cycle of pembrolizumab, which may have affected the evaluation. Nevertheless, as mentioned above, our results still add evidence to the literature on the effectiveness of pembrolizumab for the treatment of unresectable PPLELC. Regarding Patients 4 and 5, who received nivolumab, the former achieved PR, and the latter achieved SD. Evidence of nivolumab for the treatment of advanced PPLELC is limited to case reports (12, 33, 34). One case report in our study was for Patient 4, who was the first patient worldwide to respond favorably (PR) to nivolumab. However, she unfortunately developed PD during the final month of the evaluation. Patient 5 still achieved SD.

Additionally, three patients were given concurrent treatments during the course of immunotherapy, two with anlotinib and one with nab-paclitaxel. To date, reports regarding the effectiveness of anlotinib in PPLELC remain lacking due to its rarity. Subgroup analysis from the ALTER0303 trial suggested that anlotinib could improve PFS and overall survival (OS) in patients with adenocarcinoma, and prolonged survival was shown in patients with squamous cell carcinoma (35). The same findings may apply to PPLELC, but more evidence is needed to determine whether the addition of anlotinib leads to better outcomes. For nab-paclitaxel, there is no evidence indicating its efficacy in PPLELC. The KEYNOTE-407 study reported the immune-chemotherapy combination in the treatment of untreated metastatic squamous NSCLC and concluded that longer OS and PFS could be achieved when pembrolizumab was added to chemotherapy (carboplatin plus paclitaxel or nab-paclitaxel) compared with chemotherapy alone (36). According to our results, it is likely that the combination of pembrolizumab and nab-paclitaxel may be feasible in the treatment of advanced PPLELC, but more clinical trials are needed.

This study has several limitations that should be noted. The most obvious weakness is the small sample size. Due to the rare incidence of PPLELC, only 128 patients were diagnosed with PPLELC at our center over the last decade, which is among the top hospitals nationwide, with more than 5000 newly diagnosed lung cancer cases each year. Second, immunotherapy is a relatively new treatment method. Among the 128 PPLELC patients, 5 received immune checkpoint inhibitors with different treatment regimens. Whether heterogeneity exists between the different treatments requires further exploration. Additionally, the follow-up period was restricted due to the emergence of new treatments. Multicenter studies with sufficiently long observation periods will be carried out by our team in the future to provide more convincing evidence. Finally, this study represents a descriptive investigation. Research concerning the mechanism of immune checkpoint inhibitors in PPLELC is required.

In summary, we evaluated a small number of patients and demonstrated that immune checkpoint inhibitors may be promising beneficial treatments for advanced PPLELC. Optimal treatments for this type of disease remain lacking, and large clinical trials are warranted.

## Data Availability Statement

The raw data supporting the conclusions of this article will be made available by the authors, without undue reservation.

## Ethics Statement

The studies involving human participants were reviewed and approved by Ethics Committee of West China Hospital, Sichuan University, China. The patients/participants provided their written informed consent to participate in this study. Written informed consent was obtained from the individual(s) for the publication of any potentially identifiable images or data included in this article.

## Author Contributions

ZW and XX contributed to the collection and analysis of the data. ZW drafted the manuscript. DC, KW, and WL participated in interpreting the data and revising the manuscript. BC designed the study and reviewed the drafts. All authors contributed to the article and approved the submitted version.

## Funding

This research was supported by the National Key R&D Program of China (2018YFC1311900), National Natural Science Foundation of China (91859203, 81870034, and 81871890), and the Key Program of the Department of Science and Technology, Sichuan Province, China (2017SZ0052). These funders approved the design of the study but had no role in the collection, analysis, and interpretation of the data or writing of the manuscript.

## Conflict of Interest

The authors declare that the research was conducted in the absence of any commercial or financial relationships that could be construed as a potential conflict of interest.

## Abbreviations

AC, pemetrexed plus carboplatin; AP, pemetrexed plus platinum; CT, computed tomography; DP, docetaxel plus cisplatin; EBER, Epstein-Barr encoding region; GP, gemcitabine plus cisplatin; HE, hematoxylin-eosin; IHC, immunohistochemical; MRI, magnetic resonance imaging; NSCLC, non-small cell lung cancer; OS, overall survival; PD, progressive disease; PD-1, programmed cell death-1; PD-L1, programmed death-ligand 1; PET, positron emission tomography; PFS, progression-free survival; PPLELC, primary pulmonary lymphoepithelioma-like carcinoma; PR, partial remission; RECIST, Response Evaluation Criteria in Solid Tumors; SD, stable disease; TC, paclitaxel plus carboplatin; TF, paclitaxel plus fluorouracil; TP, taxanes plus platinum; TPS, tumor proportion score.
