# Supplementary material for: Immune Checkpoint Blockade Therapy May Be a Feasible Option for Primary Pulmonary Lymphoepithelioma-like Carcinoma
Source: Front Oncol. 2021 Apr 26;11:626566. doi: 10.3389/fonc.2021.626566 (PMC8110193; doi:10.3389/fonc.2021.626566)
Supplement: Supplementary file 1 [file DataSheet_1.docx]

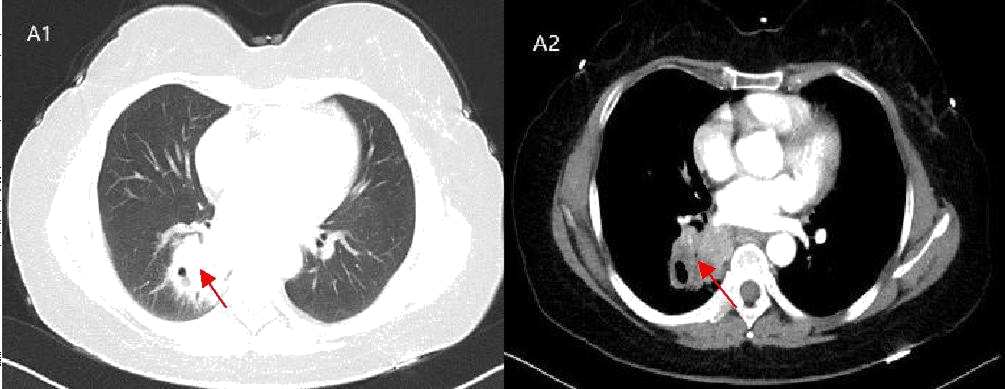
Baseline Sep, 2016


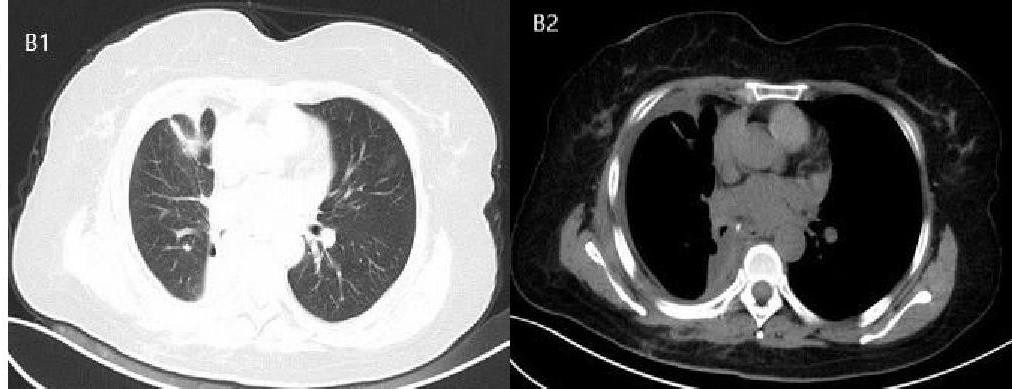
1 month after surgery

Oct, 2016


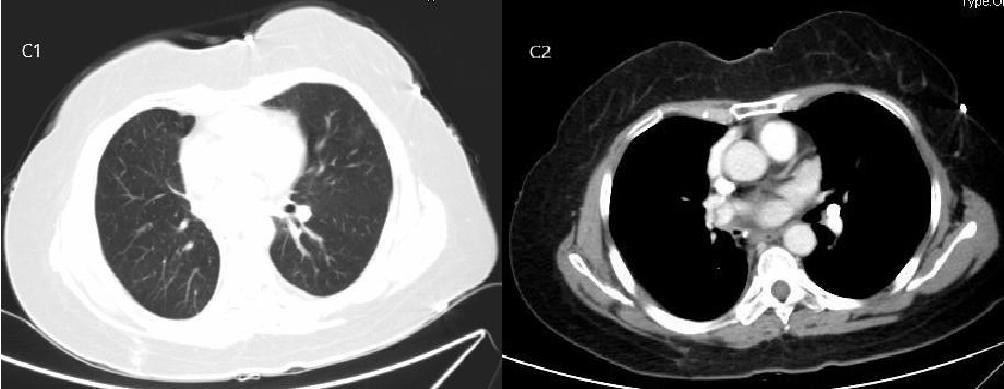
7 months after GP regimen

Sep, 2017


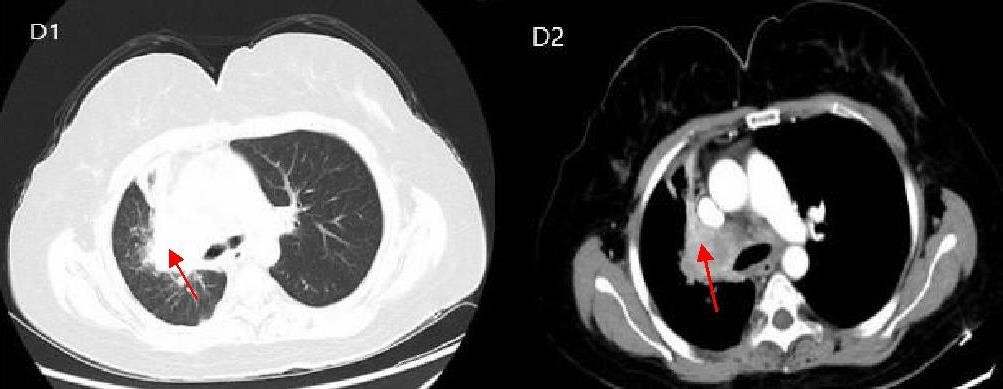
15 months after GP regimen

PD


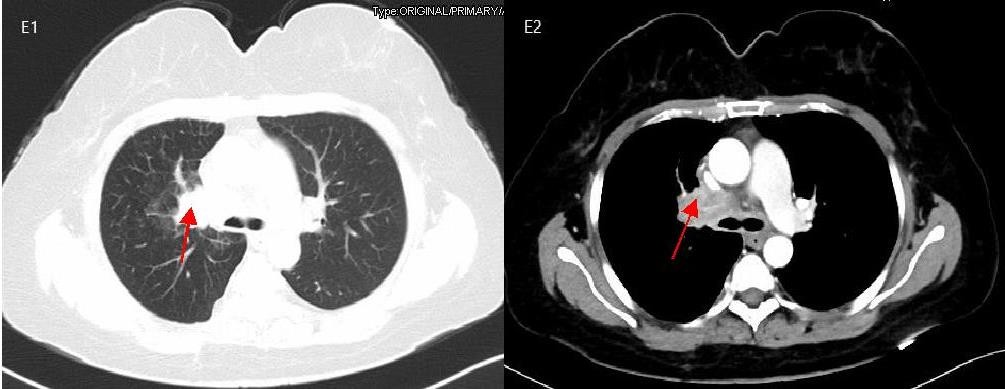
2 cycles of TC regimen

SD

July, 2018


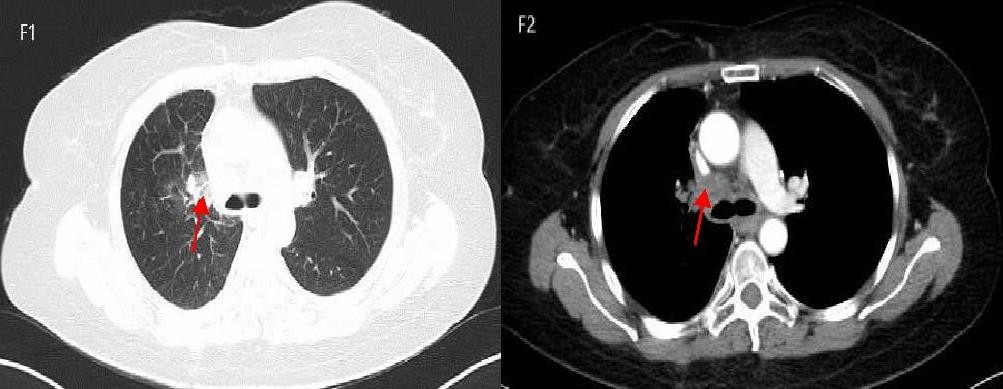
4 cycles of TC regimen

Aug, 2018


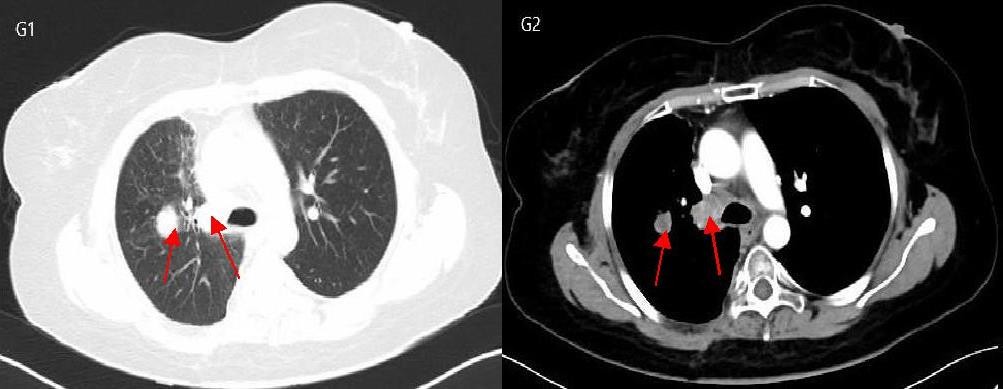
13 months after TC regimen

PD Oct, 2019


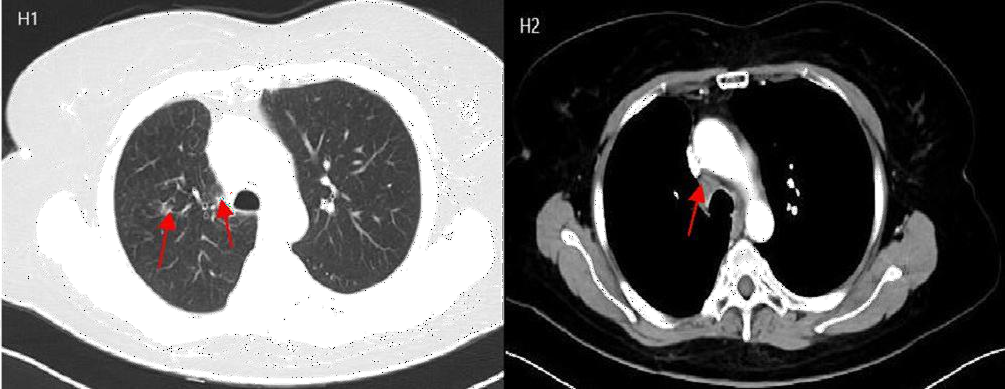
5 cycles of Sintilimab

PR

March, 2020


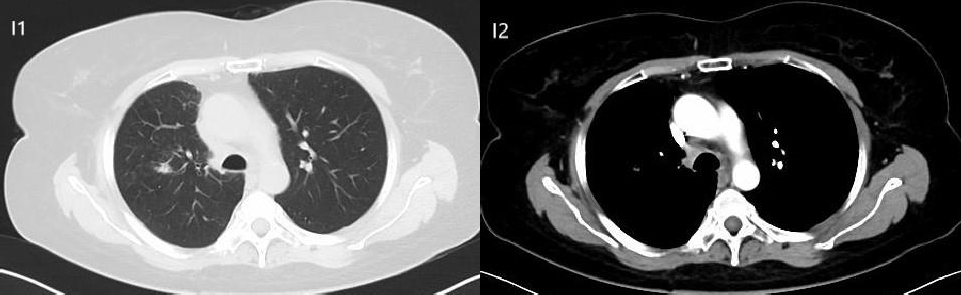
7 cycles of Sintilimab

SD

Jun, 2020

**Supplementary Figure 1.** Images of patient 1. A soft tissue mass located in right lower lobe at the baseline(A1-A2). After she underwent surgery and two cycles of GP regimen, the mass was invisible(B1-C2). On May, 2018, a new mass occurred(D1-D2). Furthermore, four cycles of TC regimen were administered and SD was achieved(E1-F2), but the tumor still progressed on Oct, 2019 with pulmonary metastasis(G1-G2). Sintilimab was given and she obtained PR after five cycles(H1-H2), SD after seven cycles (I1- I2), respectively.
