# Supplementary material for: Immune Checkpoint Blockade Therapy May Be a Feasible Option for Primary Pulmonary Lymphoepithelioma-like Carcinoma
Source: Front Oncol. 2021 Apr 26;11:626566. doi: 10.3389/fonc.2021.626566 (PMC8110193; doi:10.3389/fonc.2021.626566)
Supplement: Supplementary file 2 [file DataSheet_2.docx]

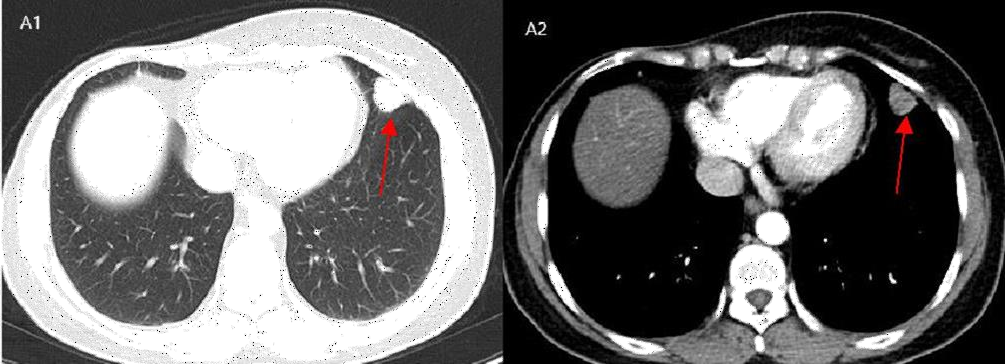
Baseline Nov, 2017


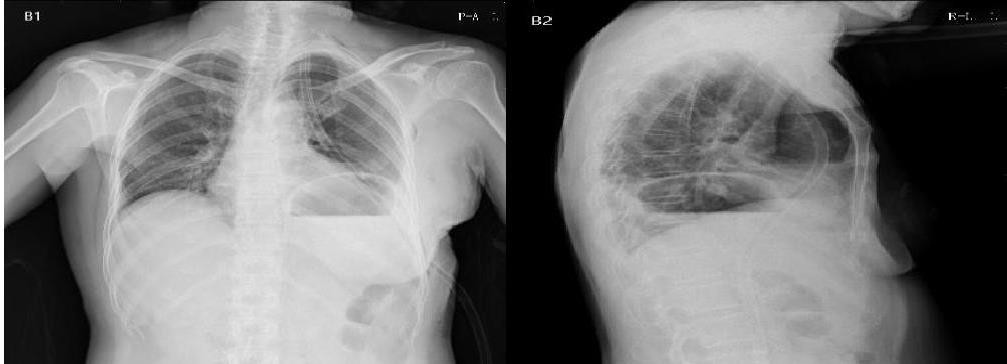
After surgery

Dec, 2017


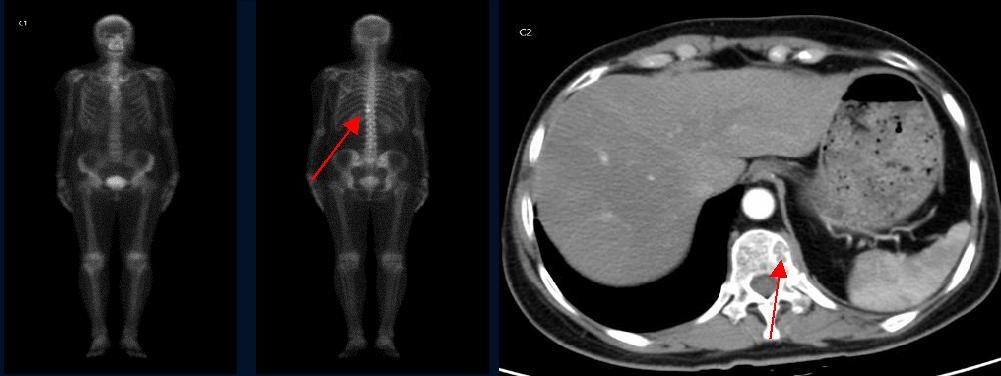
PD

Aug, 2018


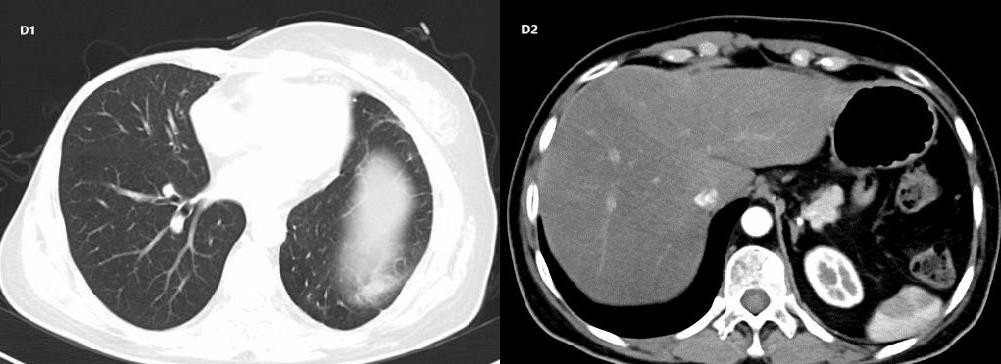
2 cycles of TC regimen+ radiotherapy

SD Oct, 2018


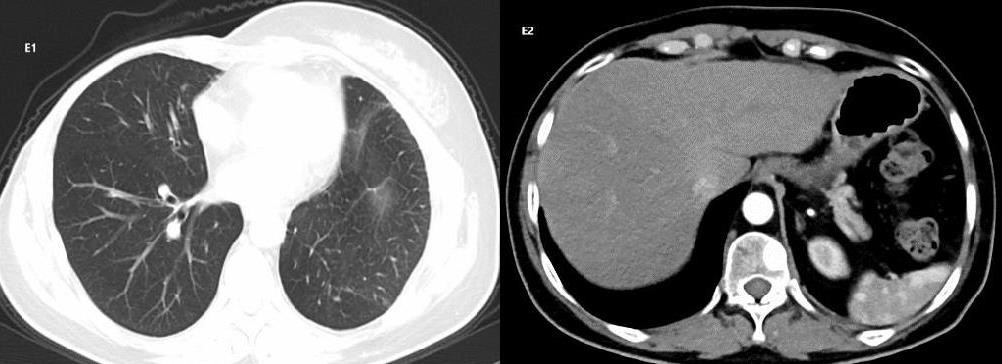
4 cycles of TC regimen

Dec, 2018


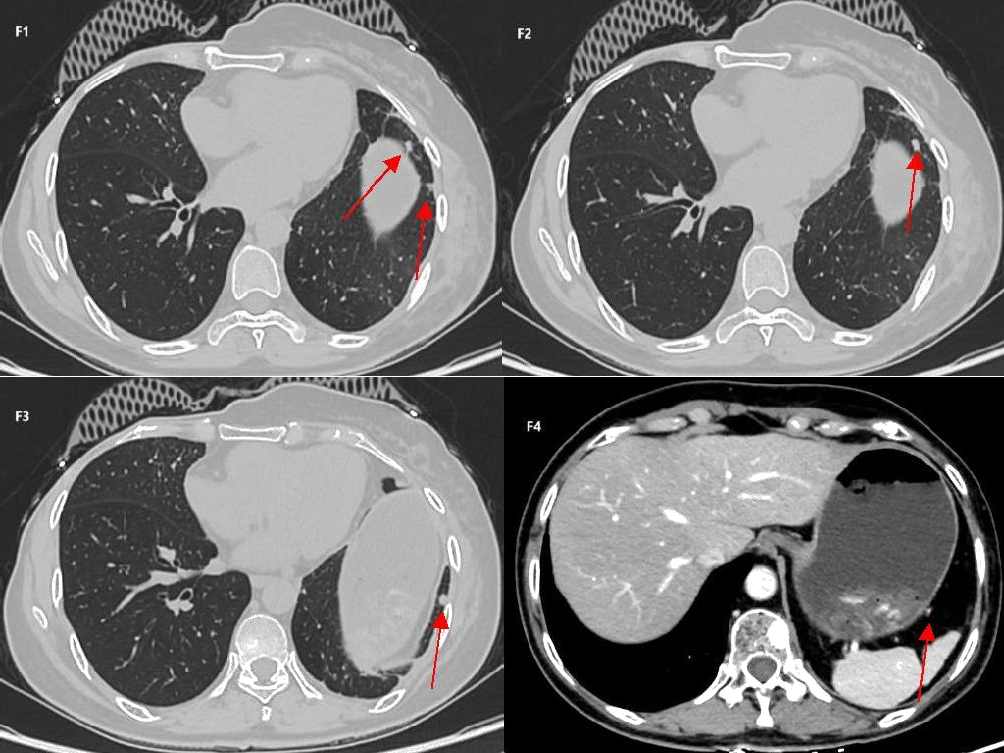
PD

July, 2019


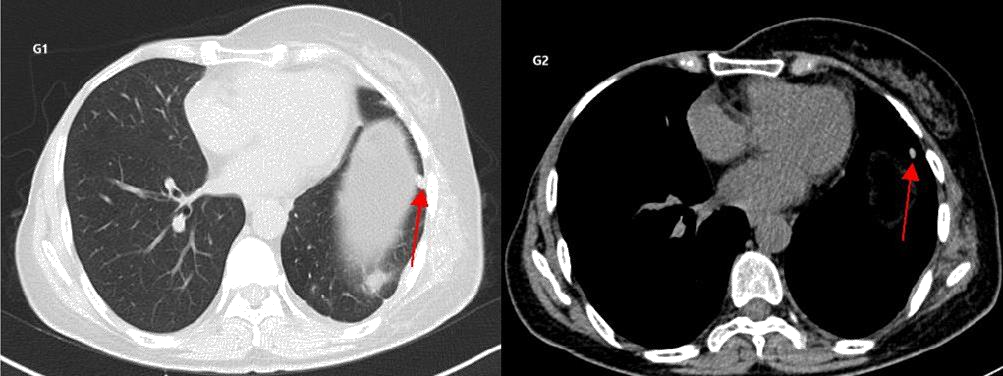
2 cycles of Pembrolizumab

SD

Sep, 2019


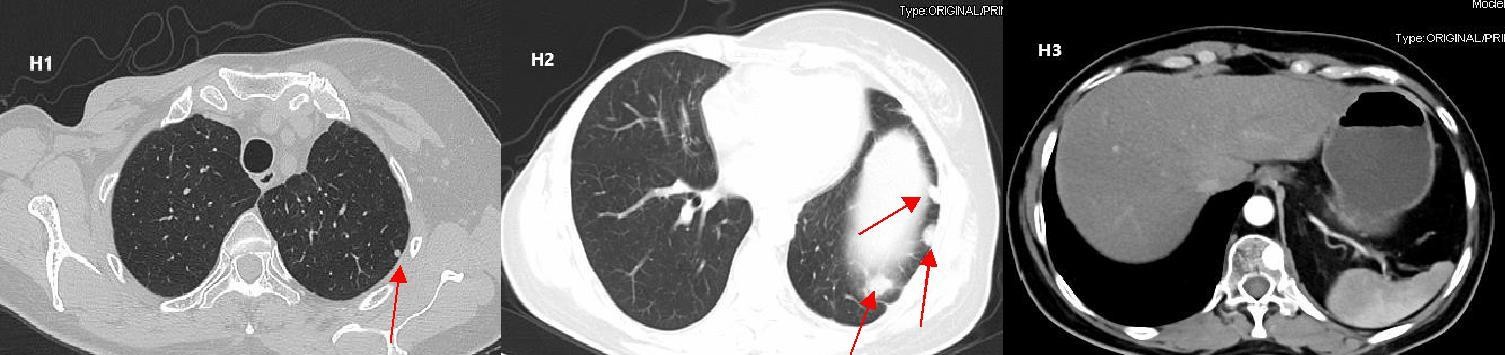
4 cycles of Pembrolizumab

PD Dec, 2019


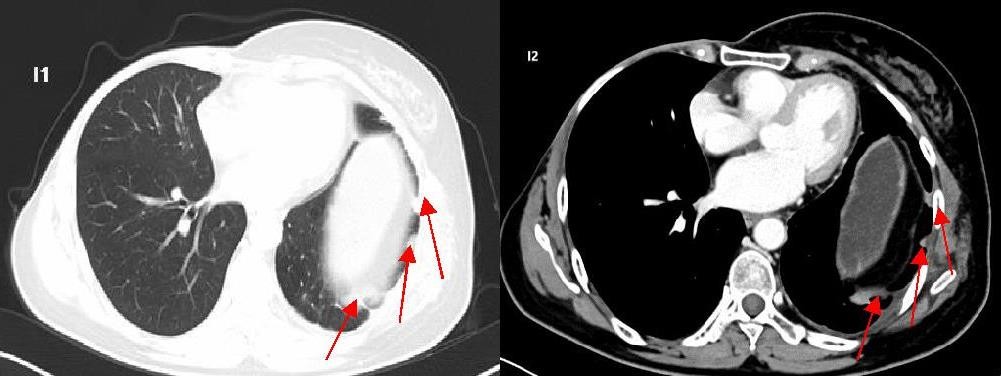
2 cycles of Pembrolizumab + Nab-paclitaxel

SD Feb, 2020

4 cycles of Pembrolizumab + Nab-paclitaxel


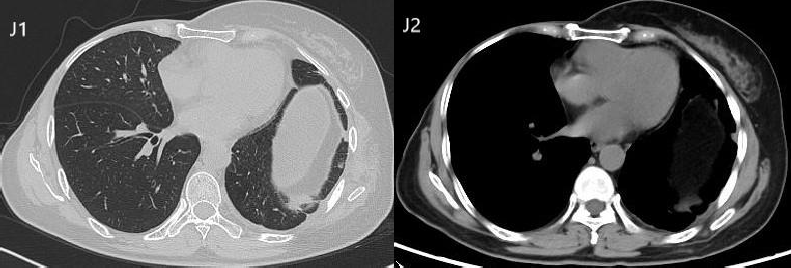

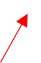


SD Feb, 2020

**Supplementary Figure 2.** Images of patient 2. A soft tissue mass located in left upper lobe at the baseline(A1-A2). She underwent surgery on 1st Dec, 2017 (B1-B2) and after eight months, there was bone metastasis (C1-C2). A total of four cycles of TC regimen plus radiotherapy were administered and SD was achieved (D1-E2). On July, 2019, pulmonary metastases in the left lower lobe were found (F1-F4). Pembrolizumab monotherapy was given initially and the nodules became larger and more(G1-H3). Afterwards, she was treated with four cycles of Pembrolizumab plus Nab-paclitaxel and presented SD.
