# Supplementary material for: Immune Checkpoint Blockade Therapy May Be a Feasible Option for Primary Pulmonary Lymphoepithelioma-like Carcinoma
Source: Front Oncol. 2021 Apr 26;11:626566. doi: 10.3389/fonc.2021.626566 (PMC8110193; doi:10.3389/fonc.2021.626566)
Supplement: Supplementary file 3 [file DataSheet_3.docx]

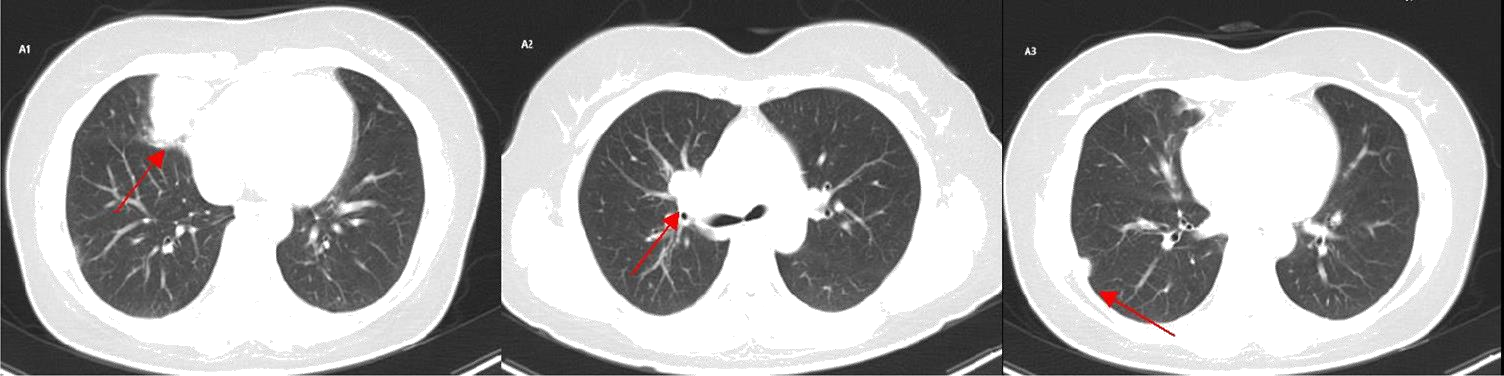

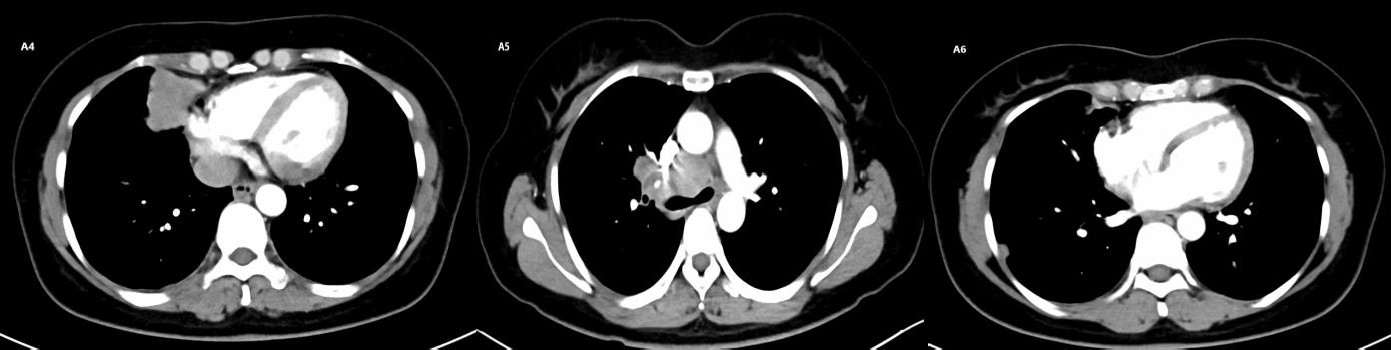

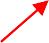

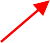

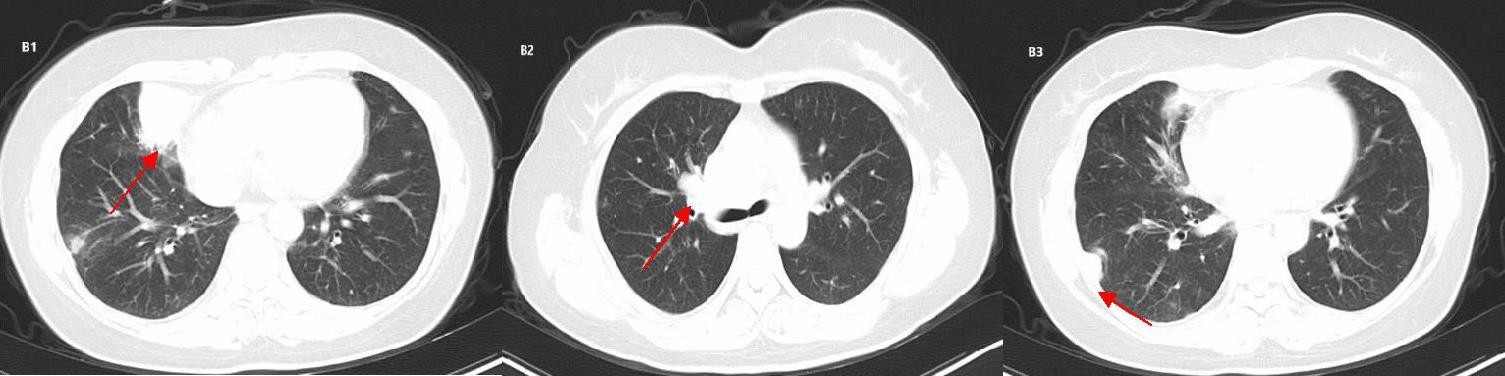

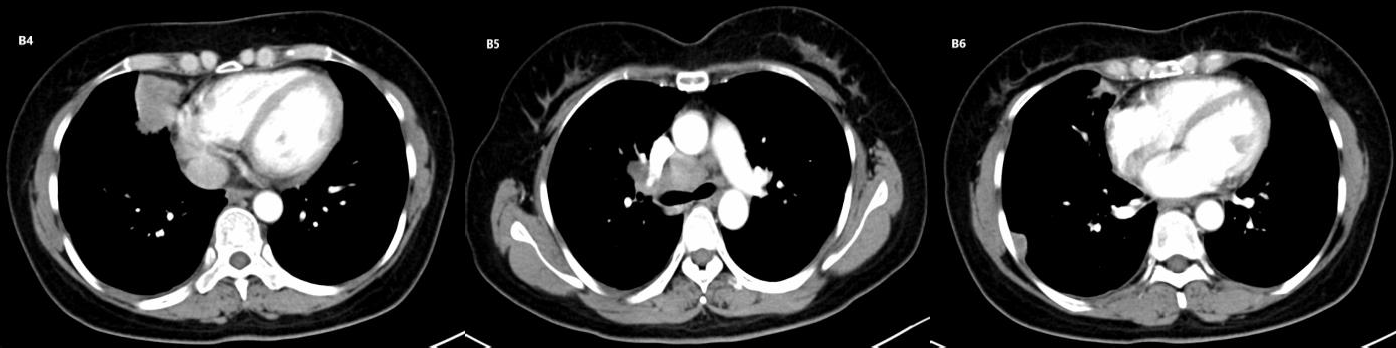

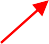

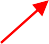


Before Pembrolizumab

July, 2018

2 months after Pembrolizumab

SD

Sep, 2018

4 months after Pembrolizumab


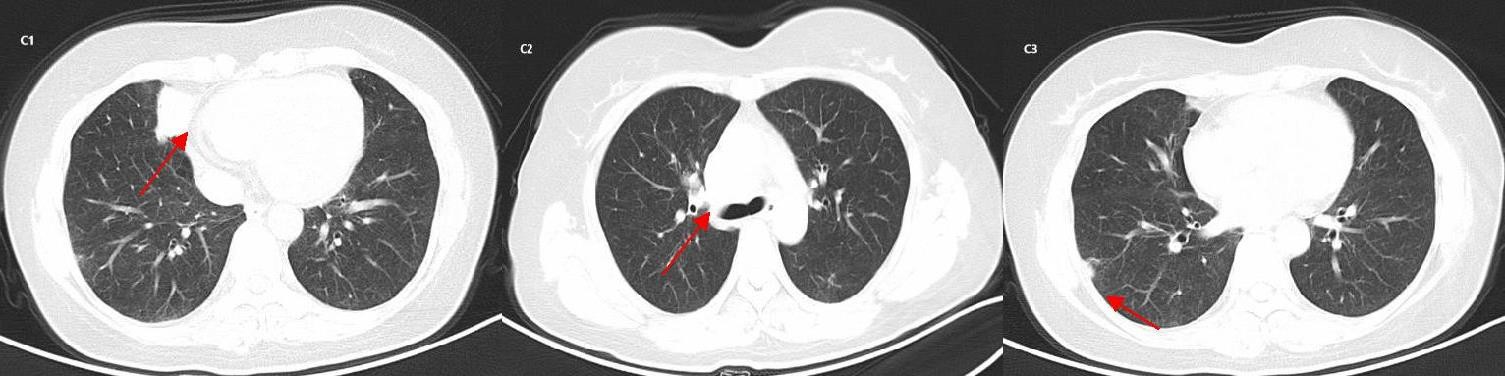

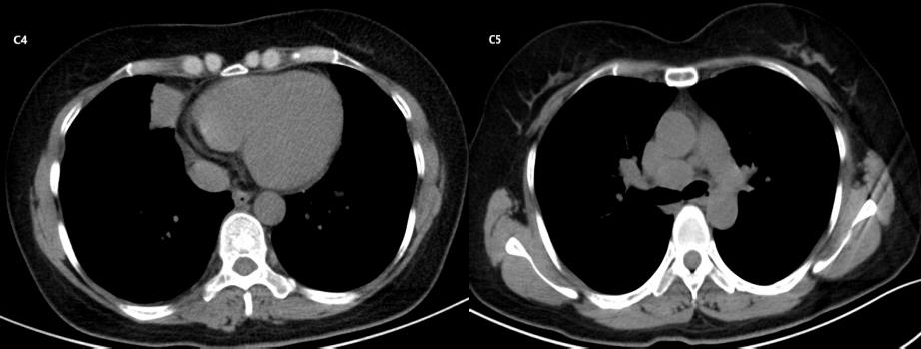

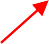

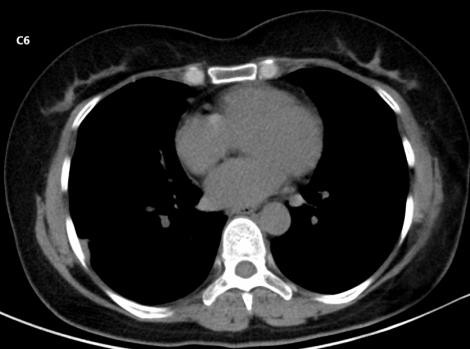

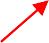


Nov, 2018

**Supplementary Figure 3.** Images of patient 3. A soft tissue mass located in right middle lobe with metastases in the mediastinal lymph nodes, right lung door as well as pleura at the baseline (A1-A6). She underwent 1 cycle of Pembrolizumab and after two months, the tumor was stable (B1-B6). Another two months passed, the mass and metastases were smaller.
