# Supplementary material for: Immune Checkpoint Blockade Therapy May Be a Feasible Option for Primary Pulmonary Lymphoepithelioma-like Carcinoma
Source: Front Oncol. 2021 Apr 26;11:626566. doi: 10.3389/fonc.2021.626566 (PMC8110193; doi:10.3389/fonc.2021.626566)
Supplement: Supplementary file 4 [file DataSheet_4.docx]

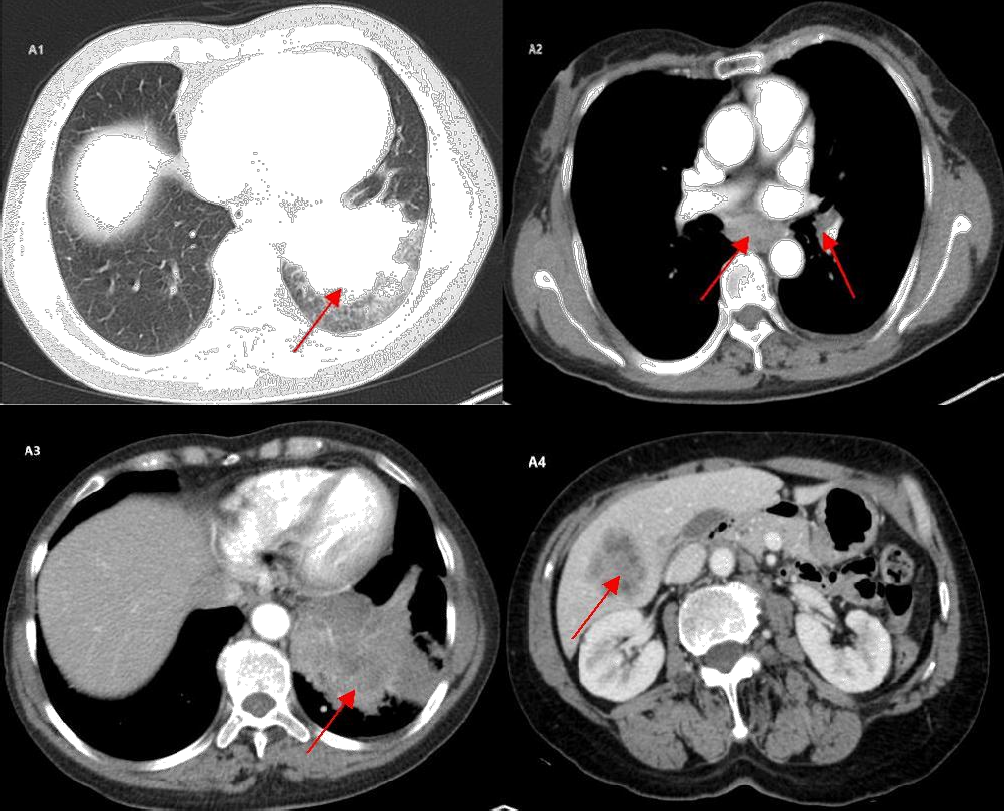
Baseline

Aug, 2018


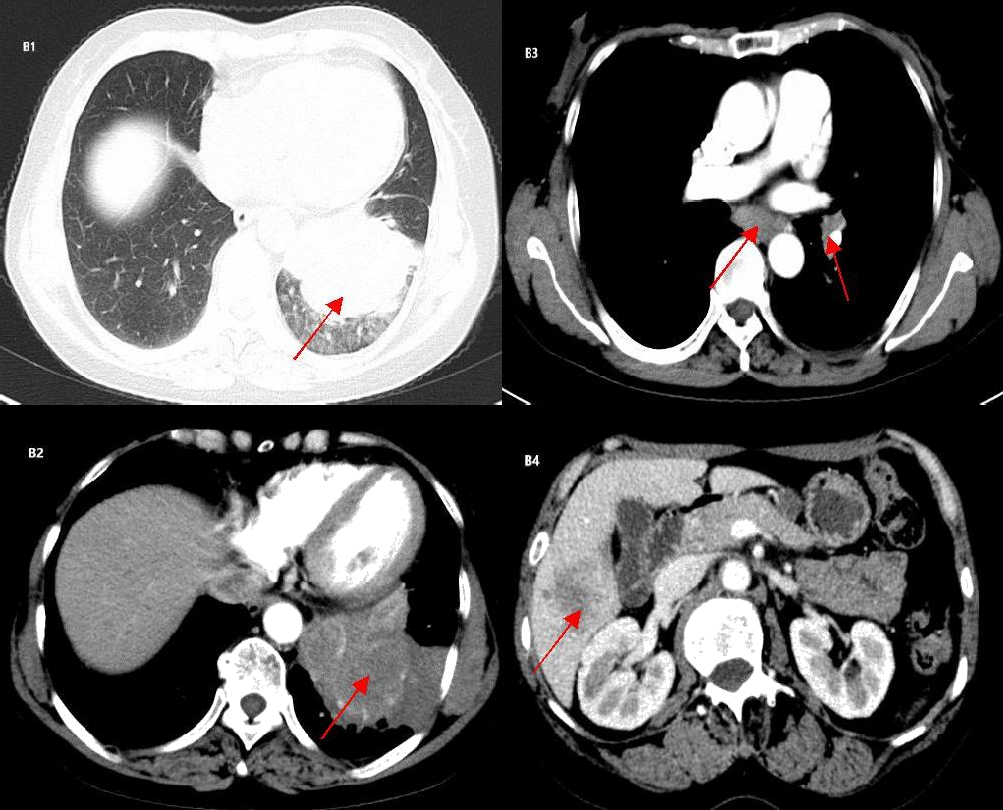
2 cycles of TC regimen

SD

Aug, 2018


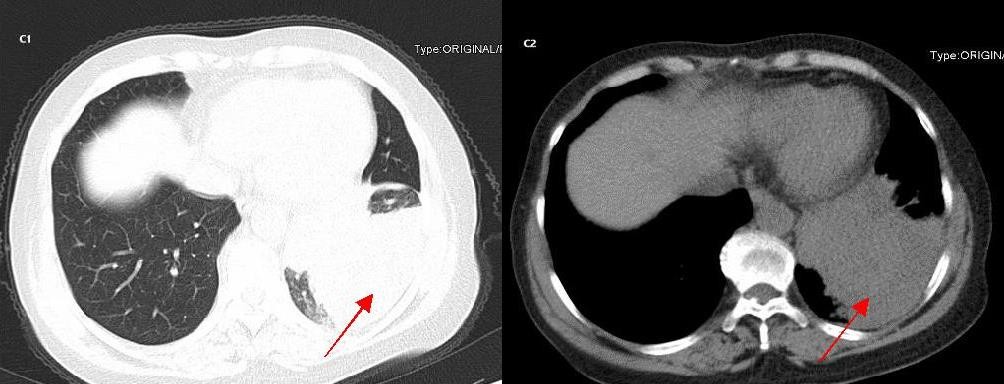
4 cycles of TC regimen

PD

Dec, 2018


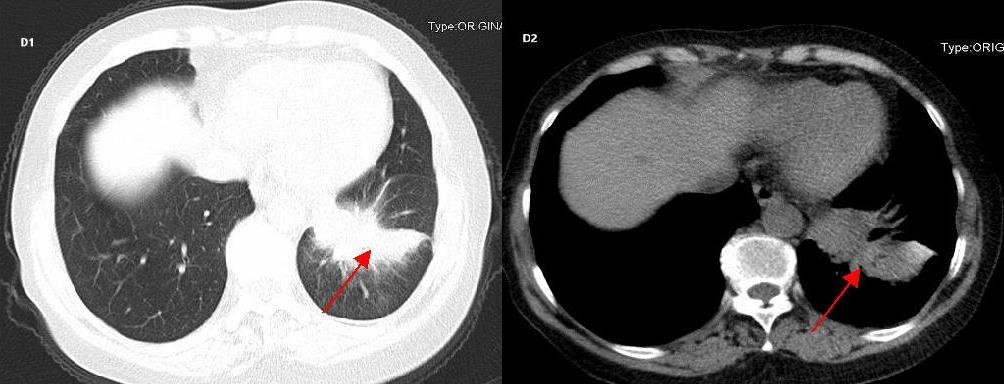
2 cycles of Nivolumab

PR Feb, 2019


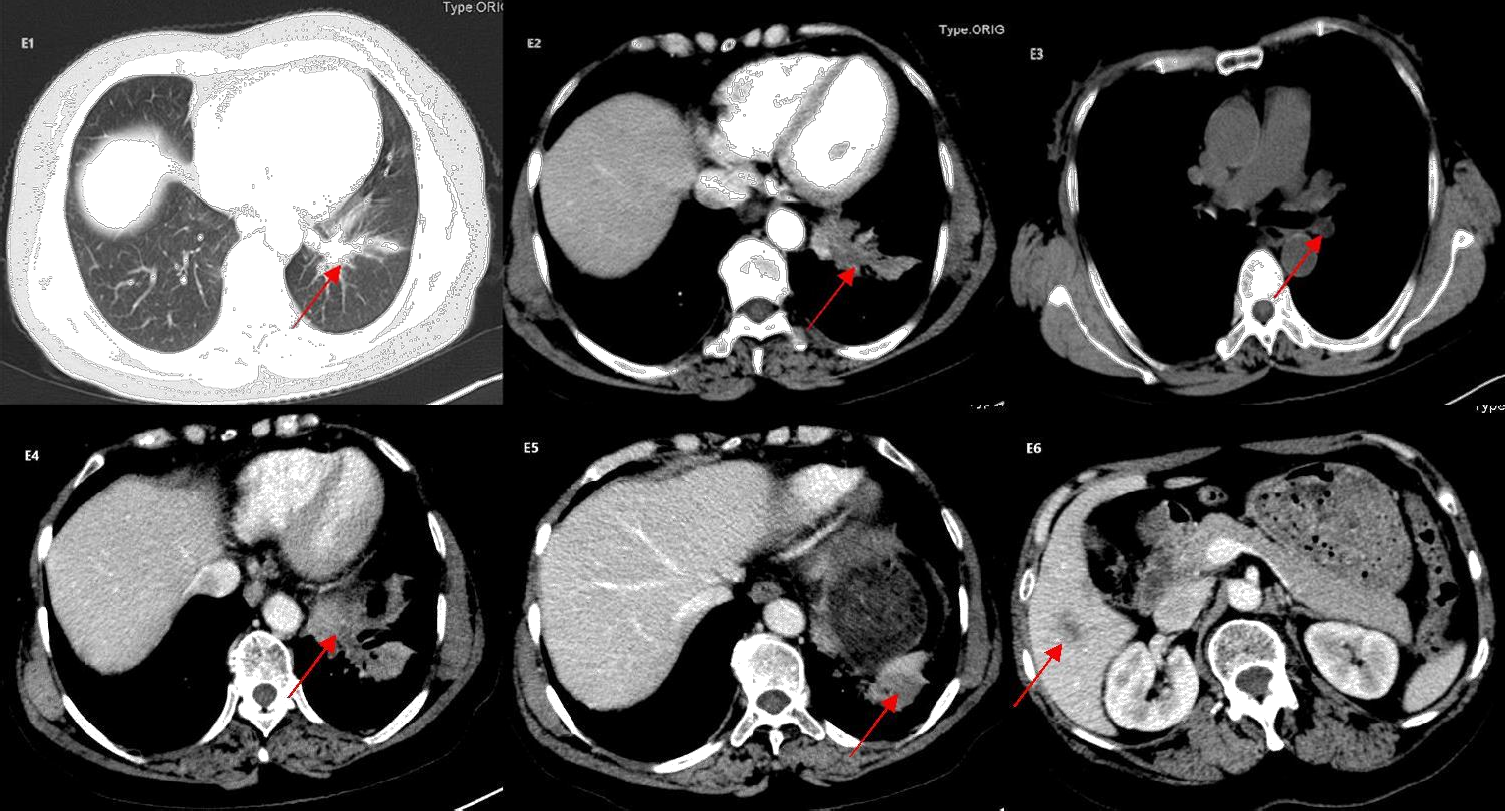
5 cycles of Nivolumab

PR

May, 2019


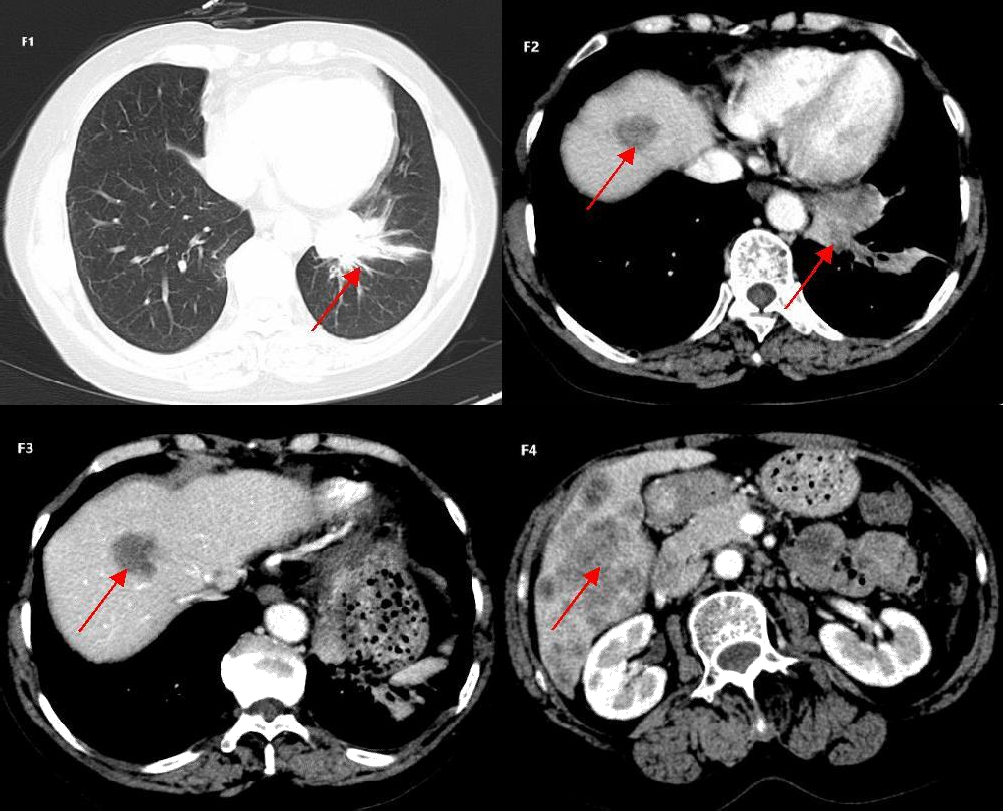
12 cycles of Nivolumab

PD

Aug, 2019

14 cycles of Nivolumab


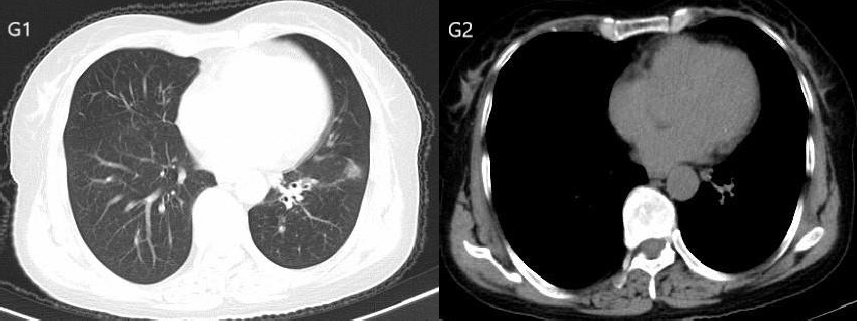

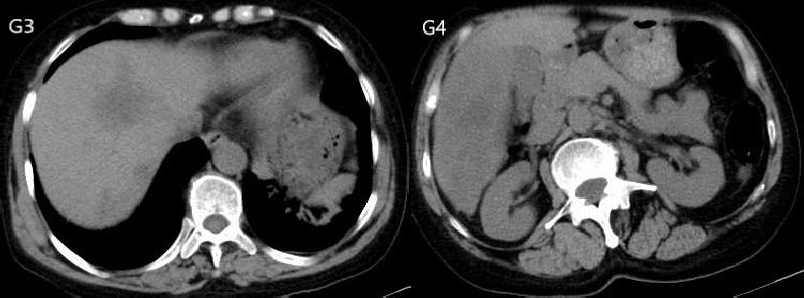


Nov, 2019

19 cycles of Nivolumab


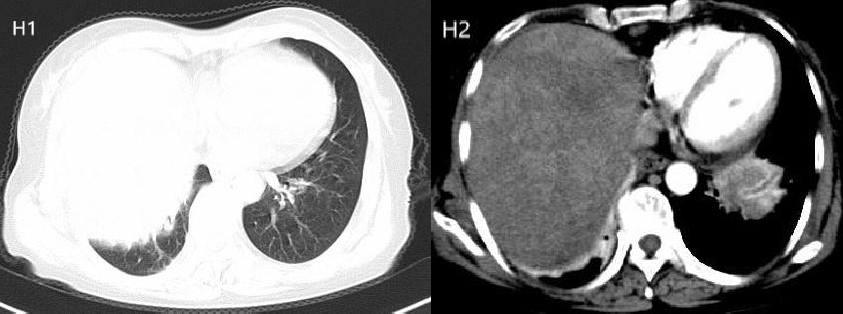


PD

Feb, 2020

**Supplementary Figure 4.** Images of patient 4. A soft tissue mass located in left lower lobe with metastases in the mediastinal lymph nodes, left lung door as well as liver at the baseline (A1-A2). Two cycle of TC regimen were given, and the tumor was stable (B1-B4). Afterwards, the mass became larger (C1-C2) after another two cycles. She presented PR to the initial five cycles of Nivolumab (D1-E6), however, after nineteen cycles, the tumor progressed (H1- H2)
