# Supplementary material for: Immune Checkpoint Blockade Therapy May Be a Feasible Option for Primary Pulmonary Lymphoepithelioma-like Carcinoma
Source: Front Oncol. 2021 Apr 26;11:626566. doi: 10.3389/fonc.2021.626566 (PMC8110193; doi:10.3389/fonc.2021.626566)
Supplement: Supplementary file 5 [file DataSheet_5.docx]

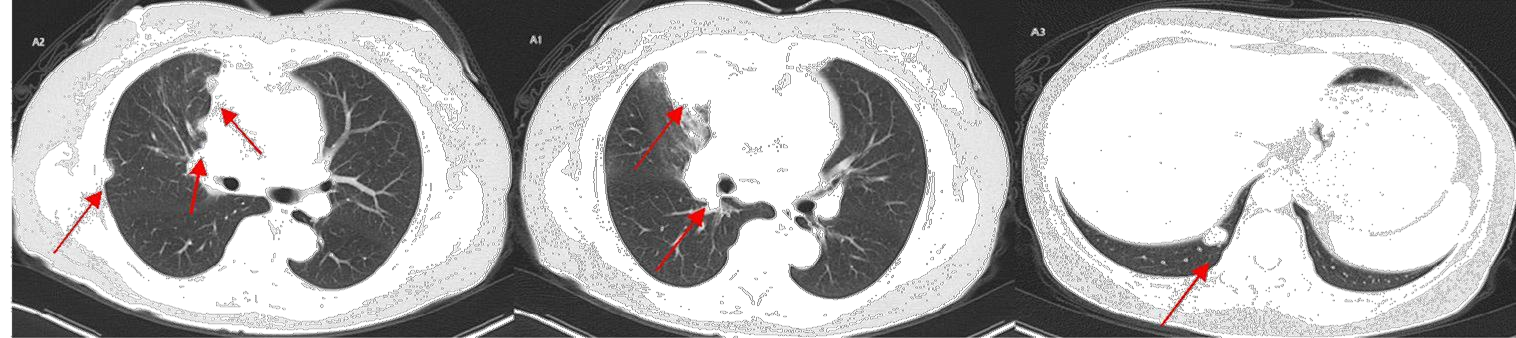
Baseline April, 2016


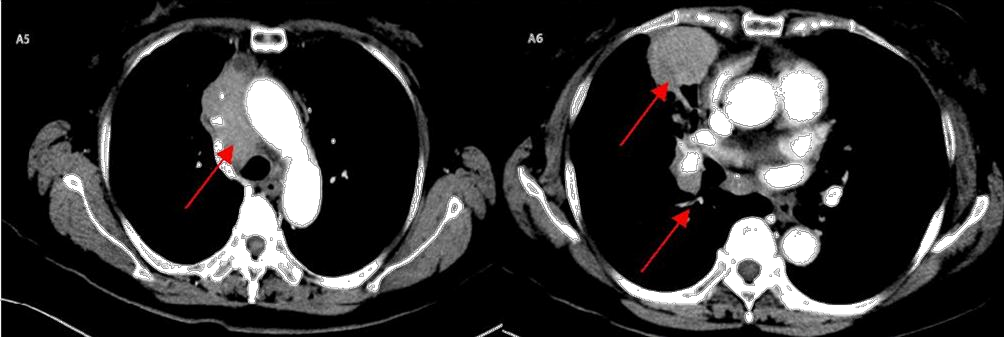


4 cycles of AC regimen


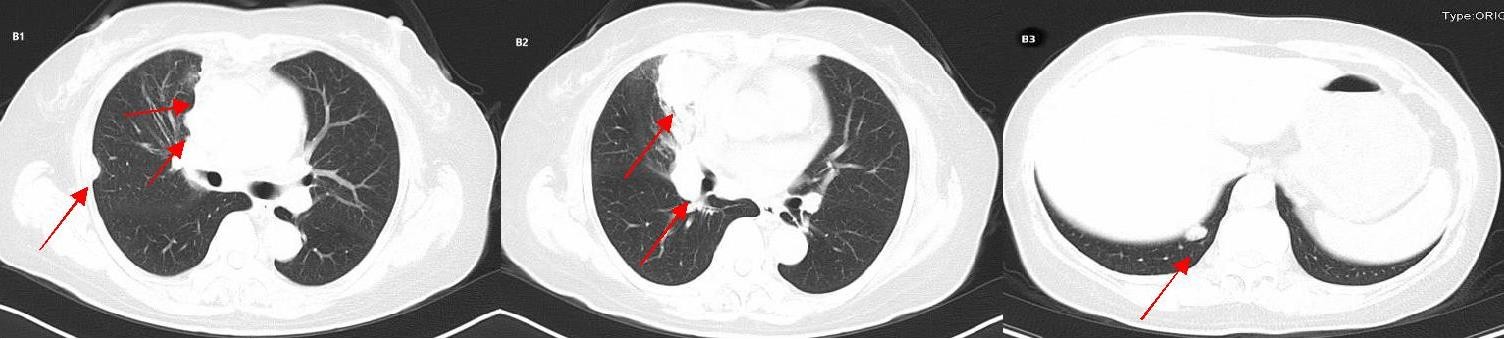
SD

Sep, 2016


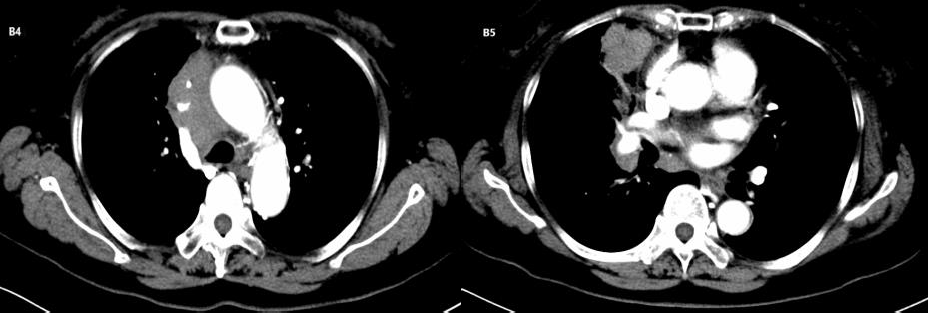


6 cycles of AC regimen


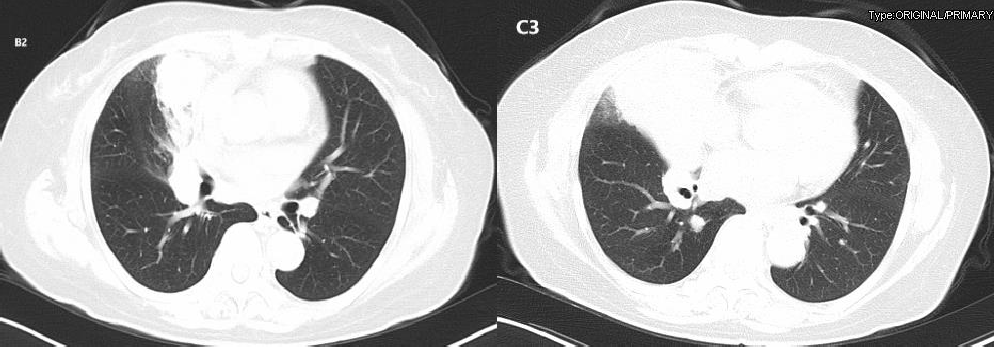

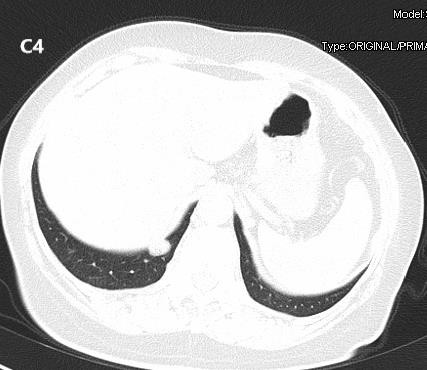

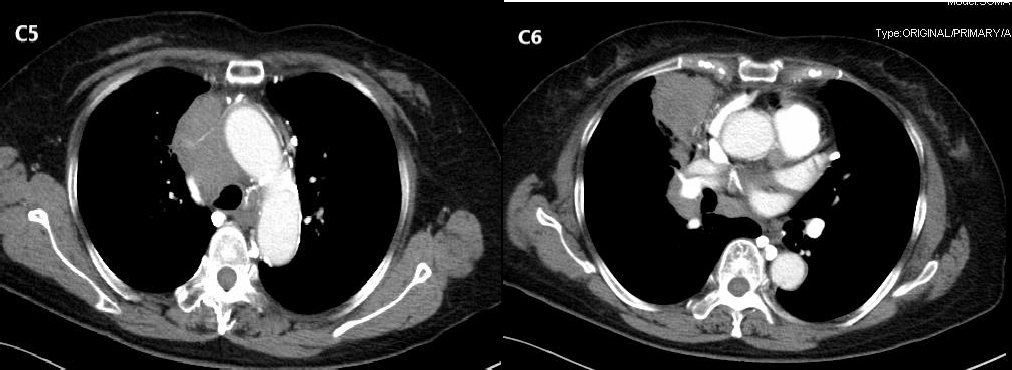

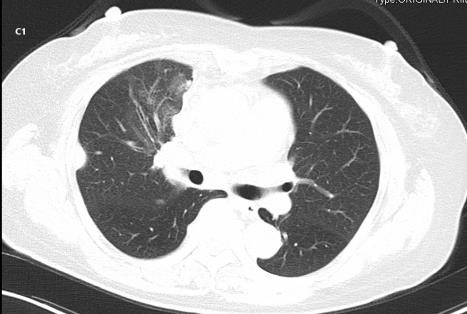


PD Feb, 2017


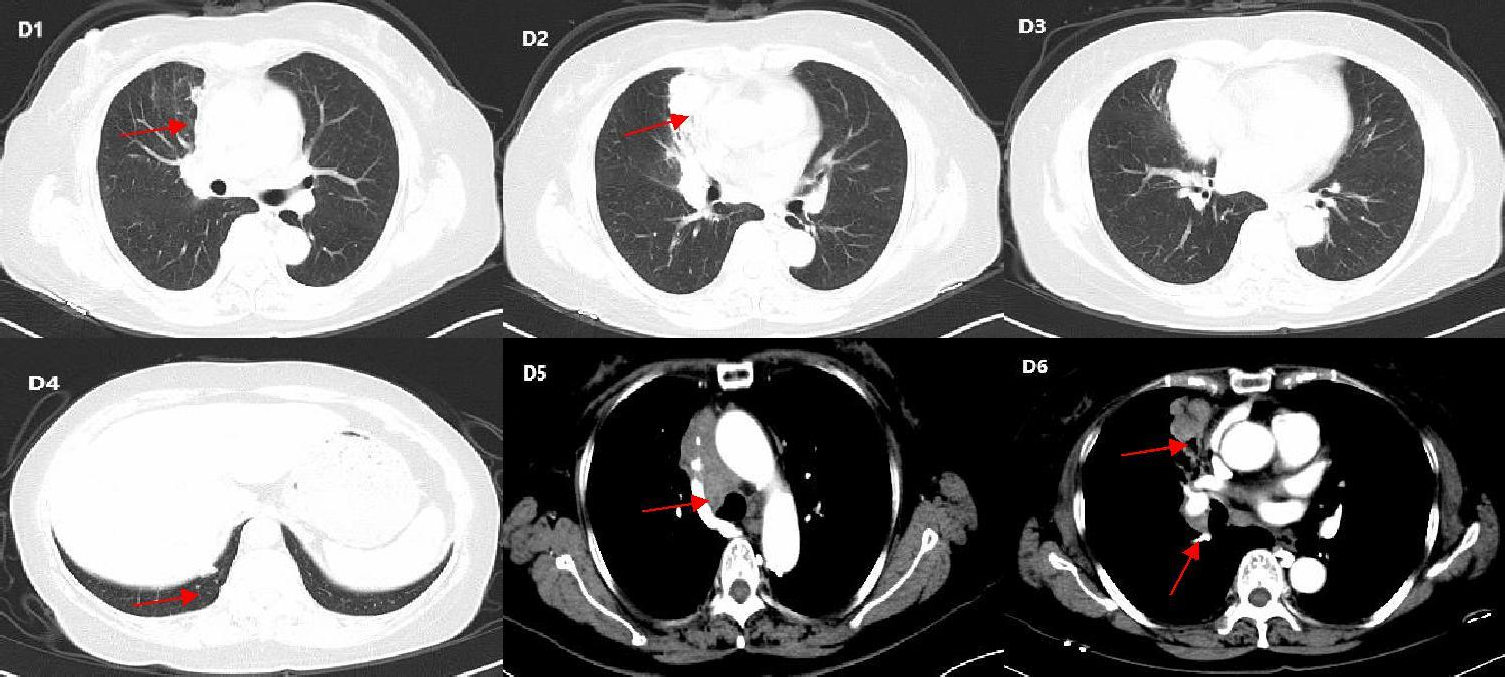
4 cycles of DP regimen

SD May, 2017

10 cycles of DP regimen


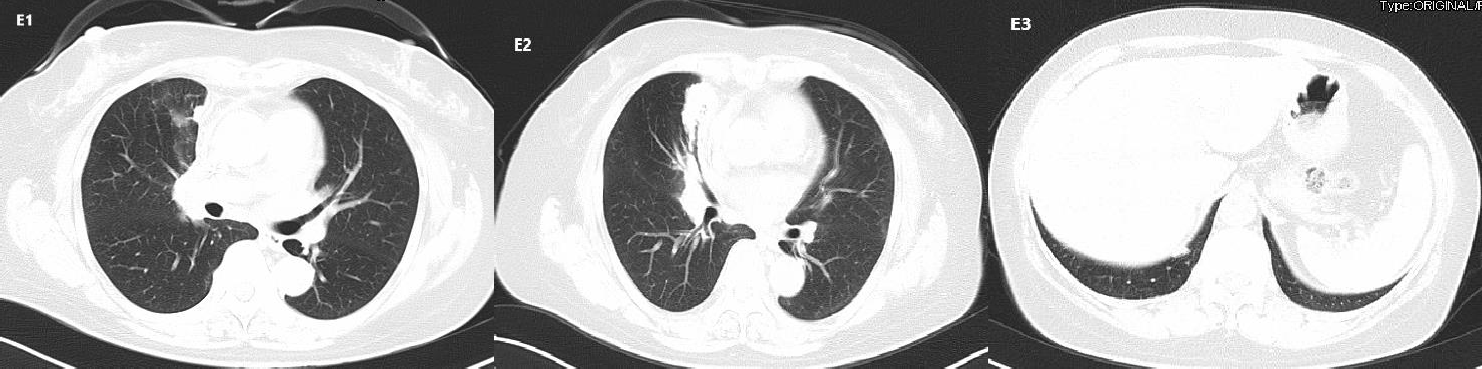

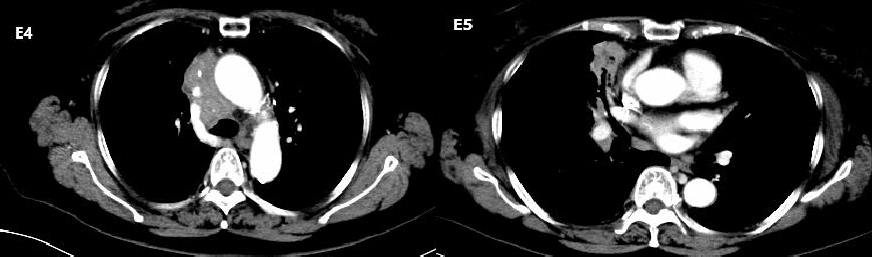

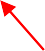

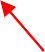

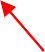

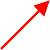


PR Sep, 2017

Two months after DP regimen


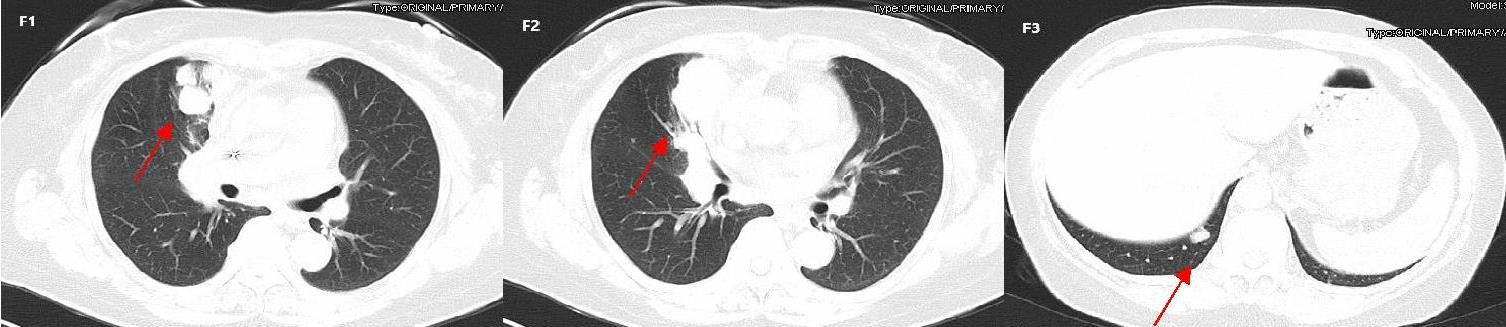

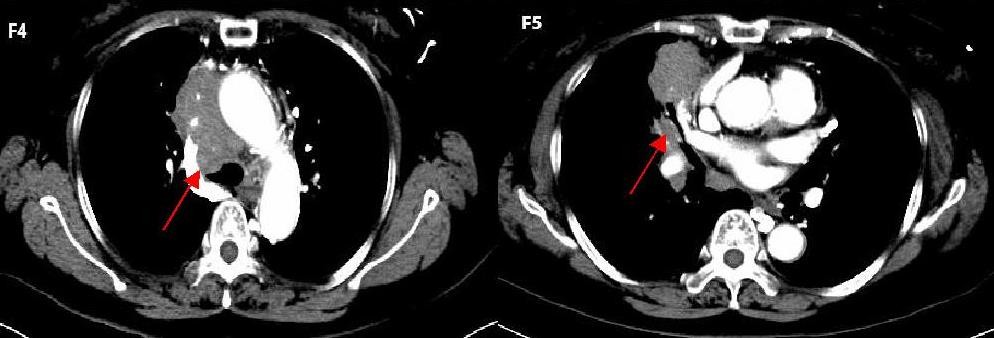


PD Dec, 2017


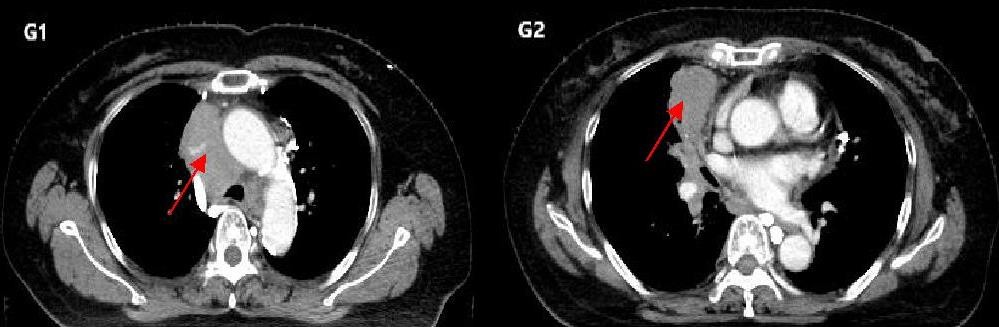
2 cycles of TF regimen

Feb, 2018

2 cycles of Nivolumab


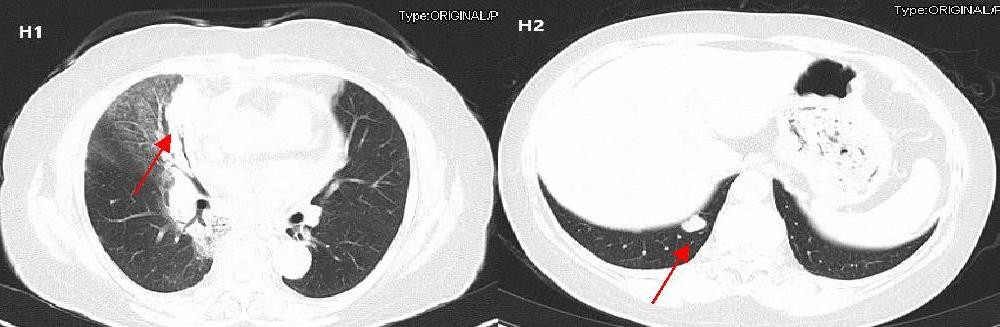

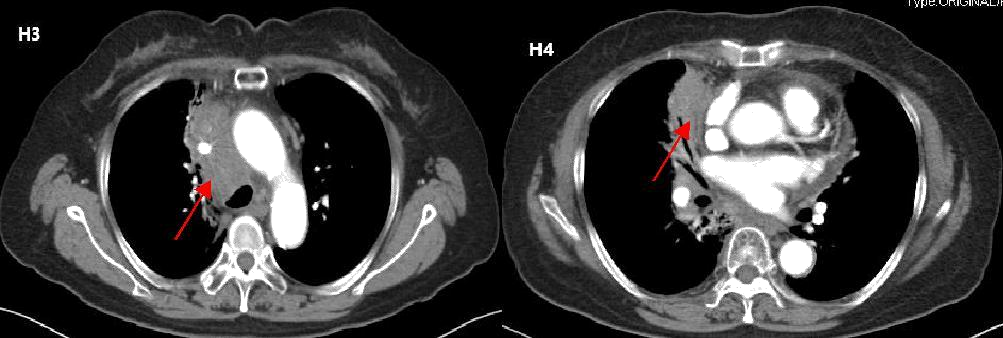


June, 2018


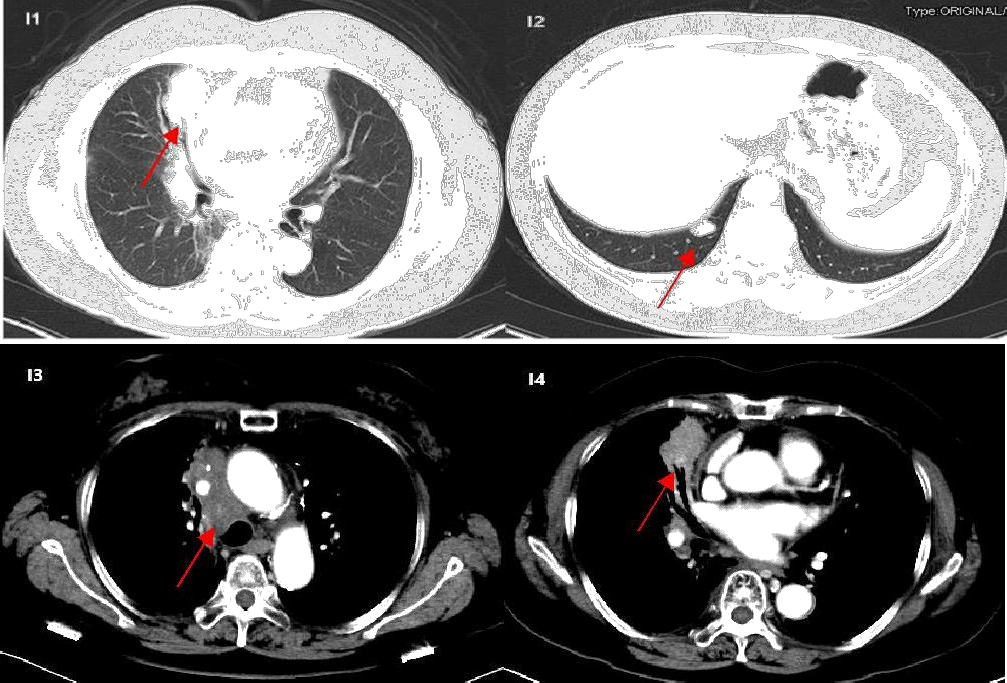
4 cycles of Nivolumab

Aug, 2018

18 cycles of Nivolumab


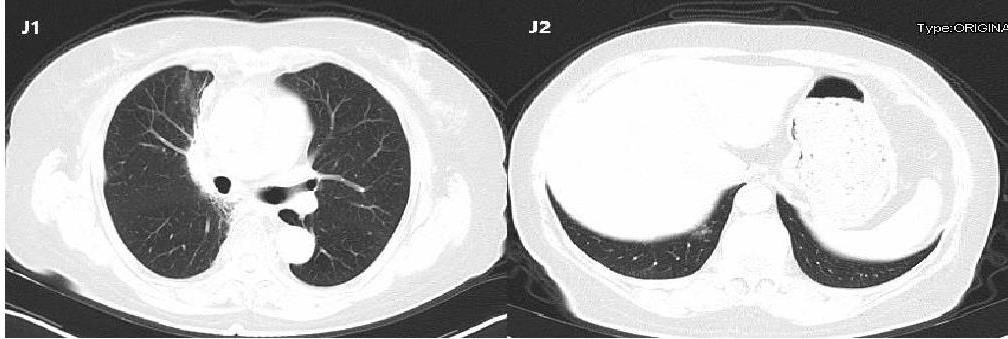

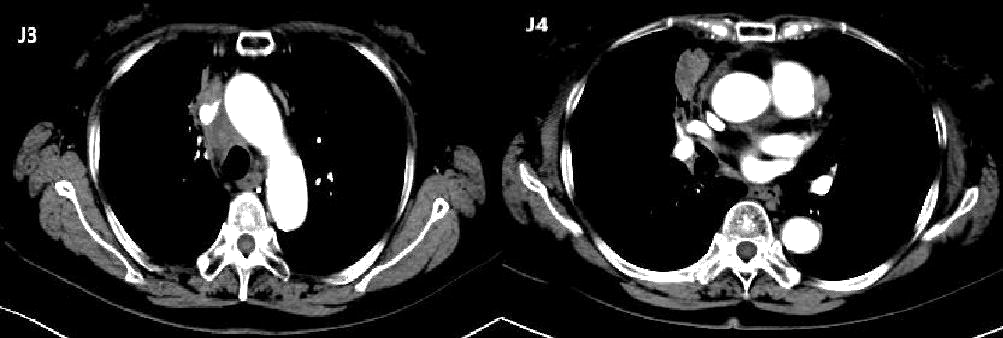


SD Oct, 2019

9 months after Nivolumab


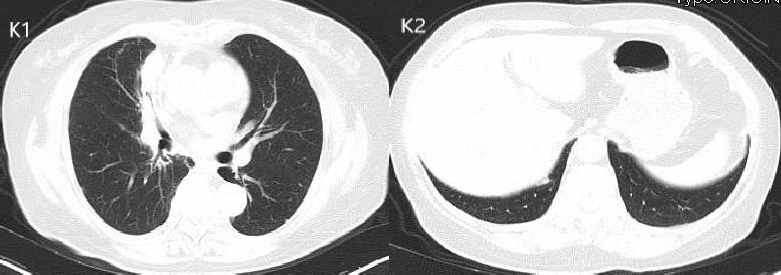


PD

May, 2020


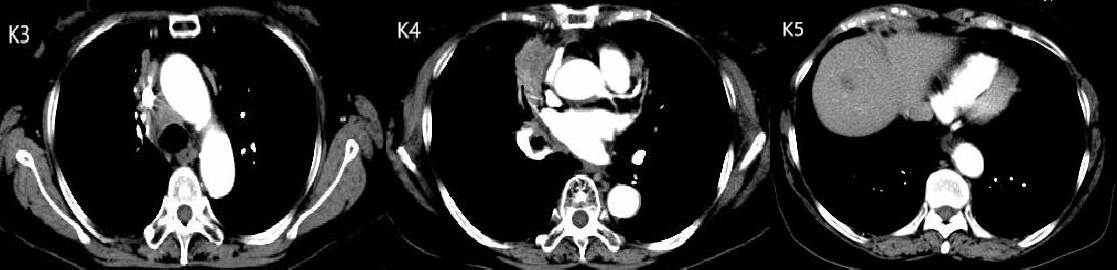


**Supplementary Figure 5.** Images of patient 5. A soft tissue mass located in right middle lobe with metastases in the right lung, mediastinal lymph nodes, right lung door as well as pleura at the baseline (A1-A6). Four cycles of AC regimen were given, and the tumor was stable (B1-B6). Another two cycles were administered and the tumor progressed with new metastasis in the right lung (C1-C6). Afterwards, 10 cycles of DP regimen were given, and PR was obtained (D1-E5), but the tumor still progressed two months later (F1-F5). She was treated with two cycles of TF regimen and eighteen cycles of Nivolumab sequentially and obtained SD (G1-J4).However, 9 months after she quitted Nivolumab, PD was presented.
