# Supplementary material for: Immune Checkpoint Blockade Therapy May Be a Feasible Option for Primary Pulmonary Lymphoepithelioma-like Carcinoma
Source: Front Oncol. 2021 Apr 26;11:626566. doi: 10.3389/fonc.2021.626566 (PMC8110193; doi:10.3389/fonc.2021.626566)
Supplement: Supplementary file 6 [file Table_1.docx]

**Supplementary Table 1. Demographic and clinical characteristics of the excluded 123 patients**

| Sex(N=123) | Male | 71(57.7%) |
| --- | --- | --- |
|  | Female | 52(42.3%) |
| Age(N=123) | <40 | 6(4.9%) |
|  | 40-59 | 80(65.0%) |
|  | ≥60 | 37(30.1%) |
| Smoking status(N=123) | Yes | 26(21.1%) |
|  | No | 97(78.9%) |
| Method of diagnosis(N=123) | Operation | 44(35.8%) |
|  | Bronchoscopy | 19(15.4%) |
|  | CT-guided percutaneous needle lung biopsy | 60(48.8%) |
| Site of tumor(N=117) | Right lung | 72(61.5%) |
|  | Right Upper lobe | 16(13.7%) |
|  | Right Middle lobe | 39(33.3%) |
|  | Right Lower lobe | 17(14.5%) |
|  | Left lung | 45(38.5%) |
|  | Left Upper lobe | 21(17.9%) |
|  | Right Lower lobe | 24(20.6%) |
| TNM staging(N=99) | T |  |
|  | 1a | 1(1.0%) |
|  | 1b | 12(12.1%) |
|  | 1c | 14(14.2%) |
|  | 2a | 12(12.1%) |
|  | 2b | 10(10.1%) |
|  | 3 | 21(21.2%) |
|  | 4 | 29(29.3%) |
|  | N |  |
|  | 0 | 40(40.4%) |
|  | 1 | 13(13.1%) |
|  | 2 | 34(34.4%) |
|  | 3 | 12(12.1%) |
|  | M |  |
|  | 0 | 78(78.8%) |
|  | 1 | 21(21.2%) |
| Overall staging(N=99) | IA | 18(18.2%) |
|  | IB | 6(6.1%) |
|  | IIA | 5(5.1%) |
|  | IIB | 13(13.1%) |
|  | IIIA | 16(16.2%) |
|  | IIIB | 16(16.2%) |
|  | IIIC | 4(4.0%) |
|  | IV | 21(21.1%) |
| Serum EBV examination(N=15) | Positive | 11(73.3%) |
|  | Negative | 4(26.7%) |
| PD-L1 expression(N=34) | <50% | 10(29.4%) |
|  | ≥50% | 24(70.6%) |
| EGFR (N=16) | Positive | 1(6.3%) |
|  | Negative | 15(93.7%) |
| ALK(N=45) | Positive | 0(0%) |
|  | Negative | 45(100%) |
| ROS-1(N=49) | Positive | 2(4.1%) |
|  | Negative | 47(95.9%) |
| Chemotherapy(N=56) | TP/TC | 28(50.0%) |
|  | GP/GC | 15(26.8%) |
|  | FP | 1(1.8%) |
|  | DP | 5(8.9%) |
|  | AC/AP | 2(3.6%) |
|  | Unclear | 5(8.9%) |
| PD(N=60) | Yes | 22(36.7%) |
|  | No | 38(63.3%) |
| Living status(N=64) | Alive | 4(6.2%) |
|  | Dead | 60(93.8%) |
